# MULTIMOORA Method-Based Schweizer–Sklar Operations for CO_2_ Geological Storage Site Selection Under Pythagorean Fuzzy Environment

**DOI:** 10.1007/s44196-023-00201-0

**Published:** 2023-03-02

**Authors:** Yang Yang, Chao Zhang

**Affiliations:** 1 School of Information Management and Engineering, Shanghai University of Finance and Economics, Shanghai, 200433 China; 2 Shanghai Key Laboratory of Financial Information Technology, Shanghai, 200433 China; 3 Research Center of Modern Service Science and Technology, Shanghai University of Finance and Economics, Shanghai, 200433 China

**Keywords:** CCUS, Site selection, Multi-criteria decision-making, MULTIMOORA method, Aggregation operator

## Abstract

The site selection of CO_2_ geological storage facilities is essential for the development of safe and efficient carbon capture, utilization, and storage (CCUS) projects. Normally, CO_2_ geological storage site selection can be regarded as a complex multi-criteria decision-making (MCDM) problem. The aim of this paper is to present an integrated decision-making method for solving the site selection problem for CO_2_ geological storage. To achieve this goal, this method is based on multi-objective optimization by ratio analysis plus the full multiplicative form (MULTIMOORA) method and prioritized aggregation operators in Pythagorean fuzzy environment. The academic contributions of this study include: first, some Pythagorean fuzzy Schweizer–Sklar prioritized aggregation (PFSSPA) operators are proposed, which take into account the priority levels of criteria and the risk preferences of decision makers. The excellent properties of these operators are given. Then this study extends the classical MULTIMOORA method based on the developed aggregation operators (named PFSSPA-MULTIMOORA), and the calculation process of this method is described in detail. Subsequently, on the basis of the constructed criteria system, the PFSSPA-MULTIMOORA method is applied to rank the alternatives. Finally, we successfully utilized the PFSSPA-MULTIMOORA method to solve the site selection problem of CO_2_ geological storage in China. A comparative analysis of existing methods verifies the effectiveness and robustness of the proposed method. This work can provide advanced decision support for researchers and practitioners.

## Introduction

Global warming and greenhouse gas (carbon dioxide, methane, nitrous oxide, etc.) emissions have emerged as the primary issues preventing the sustainable growth of human society and economic systems. According to the World Energy Outlook 2021 report released by the International Energy Agency, global coal consumption will still grow strongly in 2021, leading to the second largest increase in carbon emissions on record. The apparent discrepancy between the current global government commitment target scenario and the 2050 net-zero emissions scenario suggests that the world will need to make more demanding emission reduction commitments and stronger measures if it is to reach net-zero emissions by mid-century. Carbon capture, utilization, and storage (CCUS) technologies refer to the capture, compression, transport, and storage of carbon dioxide (CO_2_) emitted from large sources or industrial applications. This technology is considered a feasible method to reduce greenhouse gas emissions and mitigate global warming [1–3].

CO_2_ storage technology is one of the most critical steps for CCUS. Carbon sequestration involves either the diffuse removal of CO_2_ from the atmosphere, after its release, by terrestrial or marine photosynthesis and subsequent long-term storage of the carbon-rich biomass, or the capture of CO_2_ emissions at source prior to potential release, and storage in deep oceans or geological media, or through surface mineral carbonation [4]. Among them, CO_2_ geological storage is the most well-developed storage method with the lowest operating cost and the earliest large-scale integrated commercial demonstration project. CO_2_ may leak from the geological storage reservoir and harm the environment and people due to a number of circumstances, including geological conditions, engineering methods, and force majeure at the storage site [5]. Therefore, to ensure the effective operation of the CCUS project, it is particularly important to select the appropriate storage location.

In the process of site selection, it is necessary to rank candidate sites and select the best location under multiple criteria, so it can be regarded as a multi-criteria decision-making (MCDM) problem. However, it is often difficult to express oneself with accurate values due to the inherent ambiguity and uncertainty of human perception in decision-making. Therefore, Zadeh [6] proposed the fuzzy set theory to describe the uncertainty and fuzziness of realistic decision-making. With the deepening of research and exploration of unknown areas, some more advanced fuzzy sets have emerged on the basis of fuzzy set, such as intuitionistic fuzzy sets [7] and hesitant fuzzy sets [8]. When making decisions in an intuitionistic fuzzy set environment, the sum of the membership degree and non-membership degree of expert judgment is required to be less than 1, which is often not the case. To break through this limitation, Yager extended intuitionistic fuzzy set again and put forward Pythagorean fuzzy sets (PFSs) [9]. Its essence is that the sum of membership and non-membership is greater than 1, but the sum of squares is less than 1. Compared with intuitionistic fuzzy set, the Pythagorean fuzzy set is more flexible, which can describe the uncertainty of the problem more delicately and comprehensively [10]. Thus, it is of great value to use the PFSs to handle the uncertainty and fuzziness of experts’ assessment information on site selection.

In recent years, a lot of MCDM technology has been employed for the determination of optimal locations for CO_2_ geological storage [5, 11–14]. Multi-objective optimization by ratio analysis plus the full multiplicative form (MULTIMOORA) [15] is a valid MCDM method that combines the ratio system, reference point, and full multiplicative form approaches. Dominance theory is employed to obtain a final ranking based on the results of these three subordinate methods. Subsequently, considering the fuzziness and uncertainty in many decision-making processes, extended forms of the MULTIMOORA method have appeared. For example, Wu et al. [16] developed a probabilistic linguistic MULTIMOORA method with the combined weights and the improve Borda theory to solve the MCDM problems with the linguistic evaluations. Liang et al. [17] proposed an extension robust method of MULTIMOORA to support the decision of MCDM with interval-valued Pythagorean fuzzy sets. Tian et al. [18] gave an extended picture fuzzy MULTIMOORA method based on the prospect theory to handle MCDM. Application areas for these extended forms include sustainable supplier selection [19], health-care delivery quality [20], sustainable community-based tourism [21], and green development level evaluation [22]. The application of the MULTIMOORA method is also often combined with other methods. For instance, Chen et al. [23] developed an extended MULTIMOORA method, which combines the entropy weight method to obtain the objective weights of customer requirements and introduces the influence weights to prioritize quality characteristics. Based on the third-generation prospect theory and the extended MULTIMOORA method, Qin and Ma [24] proposed an emergency decision-making method integrated with interval type-2 fuzzy information. Ma et al. [25] designed the PL-MULTIMOORA method, which combines the comprehensive BWM and entropy methods to handle the waste recycling app evaluation problems. The aforementioned studies demonstrated that the classical MULTIMOORA method and its extensions are reasonable approaches for solving real-life decision problems. Therefore, it is expected to apply the MULTIMOORA method to determine optimal locations for CO_2_ geological storage.

The information aggregation operators are an important part of MCDM method. Janani et al. [26] introduced an extension from Pythagorean fuzzy sets to complex Pythagorean fuzzy sets related to weighted aggregated functions involving Einstein operator. Paul et al. [27] developed a novel multi-attribute decision-making method using advanced Pythagorean fuzzy weighted geometric operator. However, most of the existing information aggregation method are based on simple algebraic operations. Therefore, the work of building new aggregation operators is significant and challenging. In addition, the importance of criteria varies in different MCDM problems; in other words, criteria can have priority relationships. For example, in the matter of CO_2_ geological storage site selection, the priority of ‘environmental risk’ should be higher than that of ‘investment cost’ and other criteria, i.e., ‘environmental risk’ has the highest priority because the environmental impact caused by CO_2_ leakage is extremely serious. Thus, it is necessary to investigate some new aggregation operator that can reflect the priority relationships among the criteria.

The motivation of this paper is to present a novel decision-making model based on the proposed operators for CO_2_ geological storage site selection. From the perspective of correlation between criteria, the priority relationship between criteria is considered, and we propose some Pythagorean fuzzy prioritized aggregation operators using the Schweizer–Sklar operations. The original MULTIMOORA method fails to deal with the MCDM problem with Pythagorean fuzzy evaluation information. To increase the method's flexibility, we extend the original MULTIMOORA method in the Pythagorean fuzzy environment.

On the basis of the discussed, the main contributions of this paper are listed below:

1. The CO_2_ geological storage site selection is a MCDM problem. The integrity of the decision information can be maintained when evaluating each criterion for CO_2_ storage sites using Pythagorean fuzzy information, leading to more precise assessment results.
2. The aggregation operator largely affects the decision result of the MULTIMOORA method. However, most existing aggregation operators cannot consider the prioritization of criteria. To overcome this drawback, combining the PFS and PA [28] with Schweizer–Sklar t-norm and t-conorm, the Pythagorean fuzzy Schweizer–Sklar prioritized aggregation operators are proposed, and their corresponding properties are studied. The research results can help decision makers to provide theoretical support when solving the problem of CO_2_ geological storage site selection.
3. The original MULTIMOORA method has the advantages of stability and robustness compared with other MCDM methods. In this paper, we construct an extended MULTIMOORA method based on proposed aggregation operators and apply it to solve the CO_2_ geological storage site selection problem.

The remainder of this article is organized as follows. In Sect. 2, the related literature is reviewed. In Sect. 3, some theoretical foundations are reviewed, and the Pythagorean fuzzy Schweizer–Sklar prioritized aggregation operators are defined. Furthermore, an extended MULTIMOORA method based on proposed aggregation operators is constructed. In Sect. 4, a numerical example concerning the selection of CO_2_ storage sites in a Pythagorean fuzzy environment is given. Conclusions are listed in Sect. 5.

## Related Works

This section discusses the relevant literature on the information aggregation operators, the MULTIMOORA method, and the selection of CO_2_ geological storage site. In addition, we summarize the existing research gaps.

### Literature Review of Information Aggregation Operators

The information aggregation operators are one of the important concerns in the MCDM problem. They can unify the input information into a comprehensive evaluation value. Garg [29] proposed new sine trigonometric-based operational laws and defined several weighted averaging and geometric operators based on a new sine trigonometric Pythagorean fuzzy numbers. Palanikumar et al. [30] developed some new methods to solve multiple attribute decision-making problems based on Pythagorean neutrosophic normal interval-valued set. Wei and Lu [31] proposed the Pythagorean fuzzy power ordered weighted average (PFPOWA) operator and Pythagorean fuzzy power ordered weighted geometric (PFPOWG) operator to solve MCDM problems. All these existing methods described above are based on different t-norms and t-conorms, which lack flexibility in the process of aggregation.

However, it may be possible that there is a correlation between the decision-making criteria in some situations. To address such a situation, Liu et al. [32] present some new Pythagorean fuzzy linguistic Muirhead mean (PFLMM) operators to deal with MCDM problems with Pythagorean fuzzy linguistic information. Yang et al. [33] developed the Pythagorean fuzzy weighted Bonferroni mean (PFWBM) operator and the Pythagorean fuzzy weighted geometric Bonferroni mean (PFWGBM) operator. Li and Wei [34] introduced the Heronian mean and generalized the Heronian mean to provide two aggregation operators that consider the interdependent phenomena among the aggregated arguments. Xing et al. [35] proposed the Pythagorean fuzzy Choquet–Frank averaging operator and the Pythagorean fuzzy Choquet–Frank geometric operator. Jana et al. [36] constructed some Pythagorean fuzzy power Dombi operators using Dombi operations and the power averaging operator. These existing aggregation operators have not discussed the situation in which the criteria have a priority relationship among them. To tackle this problem in the Pythagorean fuzzy environment, Khan et al. [37] developed the Pythagorean fuzzy prioritized weighted average operator, the Pythagorean fuzzy prioritized weighted geometric operator and its extended forms. Gao [38] developed Pythagorean fuzzy Hamacher prioritized aggregation operators using Hamacher operations and prioritized aggregation operators.

Through the above review, aggregation operators often involve different operations, such as Hamacher, Frank, Maclaurin symmetric mean, Schweizer–Sklar and Dombi operational laws [39]. In particular, the Schweizer–Sklar t-norm and t-conorm [40] are special cases of the Archimedean t-norm and t-conorm. The property of containing parameters makes it more flexible than other operations. As an example, Biswas and Deb [41] utilized the concept of power aggregation operators through Schweizer and Sklar operations to develop Pythagorean fuzzy aggregation operators. Table 1 presents an overview of previous work on the information aggregation operators. It can be seen that there are no studies involving both the Schweizer–Sklar t-norm and t-conorm and the prioritized aggregation operators in the Pythagorean fuzzy environment. In this paper, a new Pythagorean fuzzy aggregation operator is proposed, combining the advantages of prioritized aggregation operators and the Schweizer–Sklar t-norm and t-conorm. It is valuable to note that the operators proposed in this study have the following advantages: (1) It can scientifically consider the priority relationships of criteria based on decision situations. (2) It can take the risk preference of experts into consideration based on the value of the parameter.

**Table 1:** Studies related to information aggregation operators

Author(s)	Methodology	Application
Garg [29]	Sine trigonometric Pythagorean fuzzy operators	Evaluating the five companies for investment
Palanikumar et al.[30]	Pythagorean neutrosophic normal interval-valued fuzzy aggregation operators	Selection of the personal computers
Wei and Lu [31]	Pythagorean fuzzy power aggregation operators	Evaluation of emerging technology commercialization
Liu et al. [32]	Pythagorean fuzzy linguistic Muirhead mean operators	Evaluation on the emergency response capabilities of relevant department
Yang et al. [33]	Pythagorean fuzzy Bonferroni means operators	Select the best investment option for a company
Li and Wei [34]	Pythagorean fuzzy Heronian mean operators	Supplier selection
Xing et al. [35]	Pythagorean fuzzy Choquet–Frank aggregation operators	Evaluation of service quality of domestic airline
Jana et al. [36]	Pythagorean fuzzy power Dombi operators	Investment selection
Khan et al. [37]	Pythagorean fuzzy prioritized aggregation operators	Selection of oversea teachers
Gao [38]	Pythagorean fuzzy Hamacher prioritized aggregation operators	Selection of oversea outstanding teachers
Biswas and Deb [41]	Pythagorean fuzzy Schweizer and Sklar power aggregation operators	selection of best emerging technology enterprise

### Literature Review of MULTIMOORA Method

MULTIMOORA [15] method is a valid MCDM tool that combines the ratio system, reference point, and full multiplicative form approach. The current research on the MULTIMOORA method focuses on the following two major categories, Table 2 presents an overview of previous work on the MULTIMOORA method.

**Table 2:** Studies related to MULTIMOORA method

Author(s)	Methodology	Application
Wu et al. [16]	Probabilistic linguistic MULTIMOORA	Selecting an investment brand of the karaoke television
Liang et al. [17]	IVPFPA-MULTIMOORA	Selection problem of hospital open-source EHRs systems for MedLab
Tian et al. [18]	Extended picture fuzzy MULTIMOORA method	Medical institution selection
Shang et al. [19]	Group BWM, fuzzy SEM, and MULTIMOORA	Sustainable supplier selection
He et al. [21]	Extended interval-valued Pythagorean fuzzy SWARA-MULTIMOORA	Sustainable community-based tourism
Liu et al. [42]	Intuitionistic linguistic MULTIMOORA method and intuitionistic linguistic rough MULTIMOORA method	Sustainable supplier selection
Rani et al. [43]	Fermatean fuzzy Einstein aggregation operators-based MULTIMOORA method	Location selection for electric vehicle charging stations
Irvanizam et al. [44]	Trapezoidal fuzzy neutrosophic numbers MULTIMOORA method	Cash social assistance program
Dahooie et al. [45]	The improved fuzzy MULTIMOORA method	Choosing appropriate technology forecasting method
Alkan et al. [46]	Fuzzy entropy based fuzzy COPRAS and fuzzy MULTIMOORA	Ranking of renewable energy sources for regions in Turkey
Chen et al. [47]	An extended MULTIMOORA method based on OWGA operator and Choquet integral	Determine the risk priority of failure modes
Saraji et al. [48]	An extended hesitant fuzzy set using SWARA‑MULTIMOORA method	Rank the higher education institutions

1. Combination with different fuzzy environments. Over the past few years, the MULTIMOORA method has been expanded under different fuzzy environments. For instance, Wu et al. [16] proposed a probabilistic linguistic MULTIMOORA method, and applied to the selection of shared karaoke television brands. Liang et al. [17] presented a more robust method of MULTIMOORA to solve the MCDM evaluations with interval-valued Pythagorean fuzzy sets. Tian et al. [18] gave an extended picture fuzzy MULTIMOORA method based on the PT, the MULTIMOORA method, and picture fuzzy Dice distance measures, which can be applied to MCDM problems where weight information is completely unknown. Liu et al. [42] developed an intuitionistic linguistic MULTIMOORA method and an intuitionistic linguistic rough MULTIMOORA method. Rani et al. [43] designed a decision support framework by combining MULTIMOORA method with Fermatean fuzzy sets and presented its application in electric vehicle charging station location selection. Irvanizam et al. [44] introduced a new MULTIMOORA technique with trapezoidal fuzzy neutrosophic numbers and employed it for solving group decision-making applications.
2. Combination with different MCDM techniques. MULTIMOORA method could be combined with different MCDM techniques to handle complex decision problems. For instance, Dahooie et al. [45] applied an objective weight determination method called correlation coefficient and standard deviation method to enhance the MULTIMOORA performance. Alkan et al. [46] use fuzzy complex proportional assessment and fuzzy MULTIMOORA methods to rank and evaluate renewable energy in Turkey. Chen et al. [47] proposed an extended MULTIMOORA method based on the ordered weighted geometric averaging operator and Choquet integral for failure mode and effects analysis. Shang et al. [19] designed a hybrid fuzzy decision support system by integrating MULTIMOORA method, best worst method, and fuzzy Shannon entropy method for sustainable supplier selection. He et al. [21] studied an integrated decision-making method using step-wise weight assessment ratio analysis and MULTIMOORA under interval-valued Pythagorean fuzzy sets, confirmed its applicability through an empirical case study of sustainable community-based tourism. Saraji et al. [48] developed an integrated MCDM framework, including step-wise weight assessment ratio analysis and MULTIMOORA method, and employed them for analysis and assessment of the challenges adapting the online education during the COVID-19 outbreak.

### Literature Review of CO_2_ Geological Storage Site Selection

In recent years, some academics from all around the world have dedicated their work to the field of CO_2_ geological storage site selection. Establishing a suitable methodology to select the CO_2_ geological storage site is of certain research value. For example, Guo et al. [5] introduced an extended novel TODIM method based on λ-fuzzy measure and Choquet integral to select the CO_2_ storage site using evaluation information given by decision makers which can take the form of a probabilistic hesitant fuzzy set. Hsu et al. [11] presented an effective model using the analytic network process method for selecting potential sites for CO_2_ geological storage in Taiwan. Deveci et al. [12] used fuzzy multi-criteria decision-making methods based on TOPSIS, ELECTRE, and VIKOR to assess the suitable location for CO_2_ storage in Turkey. Paul et al. [14] used a modified Pythagorean fuzzy VIKOR and DEMATEL approach for selection of CO_2_ storage site in geological media. Aviso et al. [49] developed a rough set-based machine learning approach to generate a rule-based model for the evaluation of potential CO_2_ storage sites. Raza et al. [50] presented a comprehensive screening criterion for the identification of a suitable storage site based on key properties. Through comprehensive consideration of CO_2_ geological storage technology, safety, and economic feasibility, Lu et al. [51] proposed a site ranking methodology for CGS and provided a reference for the selection of CO_2_ geological storage areas in carbonate reservoirs. Mi et al. [52] constructed an indicator system consisting of 3 indicator layers and 27 indicators. By combining the analytic hierarchy process with the fuzzy comprehensive evaluation method, the geological suitability for CO_2_ geological storage in 44 secondary tectonic units in the Junggar Basin was evaluated. Table 3 presents an overview of previous work on the CO_2_ geological storage site selection and compares it with this study.

**Table 3:** Studies related to CO_2_ geological storage site selection

Author(s)	Methodology	The priority levels of criteria
Guo et al. [5]	Extended TODIM method	×
Deveci et al. [12]	Fuzzy TOPSIS, fuzzy ELECTRE I, and fuzzy VIKOR	×
Paul et al. [14]	Modified Pythagorean fuzzy VIKOR and DEMATEL approach	×
Hsu et al. [11]	Analytic network process	×
Aviso et al. [49]	A rough set-based machine learning technique	×
Raza et al. [50]	A comprehensive screening criterion	×
Lu et al. [51]	A site ranking methodology for CO_2_ geological storage	×
Mi et al. [52]	Analytic hierarchy process and fuzzy comprehensive evaluation method	×
This study	PFSSPA-MULTIMOORA method	√

In the former research, the priority levels of criteria were not given attention, which would lead to biased decision results. The PA operator [28] is an aggregation tool that converts priority levels of criteria into weights, and it has been used in a variety of decision-making domains. It is very reasonable and valid to utilize the PA operator to reflect the priority levels of criteria regarding CO_2_ geological storage site selection.

### Research Gaps

Following conclusions could be drawn from the analysis of the above literature review.

First, despite the fact that many decision-making methods have been successfully applied in the complex CO_2_ geological storage site selection process, no research has been conducted on the PFSs-based MCDM model for selecting the best storage site. To solve this problem, this study employs the PFSs to depict the ambiguity and vagueness in the site selection process.

Second, the information aggregation operators play an important role in decision-making frameworks. It is a procedure that combines all of the individual input data into a single aggregated data set. It can be seen that the existing aggregation operators fail to capture the priority relationship among criteria, and the priority relationship among multiple criteria is extremely important in real-world decision-making. Therefore, this study proposes a new aggregation operator to make up for the above defects.

Third, the MULTIMOORA is a flexible method because it combines three powerful methods, such as the ratio system, the reference point approach, and the full multiplicative form to make ranking decisions. The classical MULTIMOORA method has been extended to different fuzzy environments. However, the classical MULTIMOORA method and its extended form fail to handle the CO_2_ geological storage site selection problems with the Pythagorean fuzzy environment. In view of the above-mentioned facts, we extend the MULTIMOORA method and the proposed operators into the Pythagorean fuzzy environment and construct a new methodology to aid site selection processes in MCDM.

## Methodology

In this section, we first introduce some fundamental theories about the Pythagorean fuzzy sets, the prioritized average operator, and the Pythagorean fuzzy Schweizer–Sklar operations. Then considering the PA operator can effectively reflect the internal connection between criteria, the Pythagorean fuzzy Schweizer–Sklar prioritized weighted average (PFSSPWA) operator and Pythagorean fuzzy Schweizer–Sklar prioritized weighted geometric (PFSSPWG) operator are developed, and their corresponding properties are proved. Finally, the MULTIMOORA method is extended to compare and rank the alternative sites.

### Theoretical Background

#### Pythagorean Fuzzy Sets

Pythagorean fuzzy sets (PFSs) [9, 53], as an extension of intuitionistic fuzzy sets, are widely used to deal with complex uncertainty in a variety of decision situations. Its basic concepts are as follows.

##### Definition 1

[9, 53]. Let $X$ be a fixed set. A PFS is an object having the form.

1 $$A=\left\{\left\langle x,{\mu }_{A}\left(x\right),{\nu }_{A}\left(x\right)\right\rangle \left|x\in X\right.\right\},$$

where ${\mu }_{A}:X\to \left[0,1\right]$ denotes the degree of membership and ${\nu }_{A}:X\to \left[0,1\right]$ denotes the degree of non-membership of element $x\in X$ to $A$, and for each $x\in X$, the following condition holds:

2 $${{\mu }_{A}}^{2}\left(x\right)+{{\nu }_{A}}^{2}\left(x\right) \leqslant 1.$$

For any PFS $A$ and $x\in X$, ${\pi }_{A}\left(x\right)=\sqrt{1-{\mu }_{A}^{2}\left(x\right)-{\nu }_{A}^{2}\left(x\right)}$ is called the degree of hesitancy of $x$ to $A$. Moreover, $A=\langle {\mu }_{A}\left(x\right),{\nu }_{A}\left(x\right)\rangle$ are the Pythagorean fuzzy numbers (PFNs), which are denoted by $A=\langle {\mu }_{A},{\nu }_{A}\rangle$, where ${\mu }_{A}\in \left[0,1\right]$, ${\nu }_{A}\in \left[0,1\right]$, ${\pi }_{A}=\sqrt{1-{\mu }_{A}^{2}-{\nu }_{A}^{2}}$, and ${{\mu }_{A}}^{2}+{{\nu }_{A}}^{2} \leqslant 1$.

##### Definition 2

[9] Let $\alpha =\langle {\mu }_{\alpha },{\nu }_{\alpha }\rangle$,${\alpha }_{i}=\langle {\mu }_{{\alpha }_{i}},{\nu }_{{\alpha }_{i}}\rangle \left(i=1,2,\dots ,n\right)$ be some PFNs; then the following operations are given:

- $${\alpha }_{1}\oplus {\alpha }_{2}=\left\langle \sqrt{{\mu }_{{\alpha }_{1}}^{2}+{\mu }_{{\alpha }_{2}}^{2}-{\mu }_{{\alpha }_{1}}^{2}{\mu }_{{\alpha }_{2}}^{2}},{\nu }_{{\alpha }_{1}}{\nu }_{{\alpha }_{2}}\right\rangle $$.
- $${\alpha }_{1}\otimes {\alpha }_{2}=\left\langle {\mu }_{{\alpha }_{1}}{\mu }_{{\alpha }_{2}},\sqrt{{\nu }_{{\alpha }_{1}}^{2}+{\nu }_{{\alpha }_{2}}^{2}-{\nu }_{{\alpha }_{1}}^{2}{\nu }_{{\alpha }_{2}}^{2}}\right\rangle $$.
- $$\lambda \alpha =\left\langle \sqrt{1-{\left(1-{\mu }_{\alpha }^{2}\right)}^{\lambda },{\nu }_{\alpha }^{\lambda }}\right\rangle ,\lambda >0.$$
- $${\alpha }^{\lambda }=\left\langle {\mu }_{\alpha }^{2},\sqrt{1-{\left(1-{\nu }_{\alpha }^{2}\right)}^{\lambda }}\right\rangle ,\lambda >0.$$

To rank the PFNs, the score function and the accuracy function are given.

##### Definition 3

[31] Let $\alpha =\langle {\mu }_{\alpha },{\nu }_{\alpha }\rangle$ be a PFN. A score function $S$ of a PFN can be represented as follows:

3 $$S\left(\alpha \right)=\frac{1}{2}\left(1+{\mu }_{\alpha }^{2}-{\nu }_{\alpha }^{2}\right),S\left(\alpha \right)\in \left[0,1\right].$$

##### Definition 4

[54] Let $\alpha =\langle {\mu }_{\alpha },{\nu }_{\alpha }\rangle$ be a PFN. An accuracy function $H$ of a PFN can be represented as follows:

4 $$H\left(\alpha \right)={\mu }_{\alpha }^{2}+{\nu }_{\alpha }^{2},H\left(\alpha \right)\in \left[-1,1\right].$$

##### Definition 5

[54] Let ${\alpha }_{i}=\langle {\mu }_{{\alpha }_{i}},{\nu }_{{\alpha }_{i}}\rangle \left(i=1,2\right)$ be two PFNs; if $S\left({\alpha }_{1}\right)>S\left({\alpha }_{2}\right)$, then ${\alpha }_{1}>{\alpha }_{2}$; if $S\left({\alpha }_{1}\right)=S\left({\alpha }_{2}\right)$, then ① if $H\left({\alpha }_{1}\right)>H\left({\alpha }_{2}\right)$, then ${\alpha }_{1}>{\alpha }_{2}$; ② if $H\left({\alpha }_{1}\right)=H\left({\alpha }_{2}\right)$, then ${\alpha }_{1}={\alpha }_{2}$.

##### Definition 6

[55] Let $X$ be a fixed set and ${\alpha }_{i}=\langle {\mu }_{{\alpha }_{i}},{\nu }_{{\alpha }_{i}}\rangle \left(i=1,2\right)$ be two PFNs; then, the distance measure between two PFSs is defined as.

5 $$d\left({\alpha }_{1},{\alpha }_{2}\right)=\frac{1}{2n}\sum_{i=1}^{n}\left(\left|{\mu }_{{\alpha }_{1}}^{2}-{\mu }_{{\alpha }_{2}}^{2}\right|+\left|{\nu }_{{\alpha }_{1}}^{2}-{\nu }_{{\alpha }_{2}}^{2}\right|+\left|{\pi }_{{\alpha }_{1}}^{2}-{\pi }_{{\alpha }_{2}}^{2}\right|\right),$$

where ${\pi }_{{\alpha }_{1}}$ and ${\pi }_{{\alpha }_{2}}$ are the hesitant degrees of element $x$ belonging to ${\alpha }_{1}$,${\alpha }_{2}$.

#### Prioritized Average Operator

The PA operator [28] was first presented by Yager, and it is widely used in the information aggregation field. It considers the priority relationship between criteria, making information aggregation more scientific. The PA operator was given as follows:

##### Definition 7

Let $C=\left({C}_{1},{C}_{2},\dots ,{C}_{n}\right)$ be a set of the criterion, that there is a prioritization between the criteria expressed by the linear ordering ${C}_{1}\succ {C}_{2}\succ {C}_{3}\succ \cdots \succ {C}_{n}$, indicating that criterion ${C}_{j}$ has a higher priority than ${C}_{k}$ if $\forall j<k$. The value ${C}_{j}\left(x\right)$ is the performance of any alternative $x$ under criterion ${C}_{j}$ and satisfies ${C}_{j}\left(x\right)\in \left[0,1\right]$. The PA operator is defined as:

6 $$PA\left({C}_{j}\left(x\right)\right)=\sum_{j=1}^{n}{\omega }_{j}{c}_{j}\left(x\right),$$

where $${\omega }_{j}=\frac{{T}_{j}}{{\sum }_{j=1}^{n}{T}_{j}}$$, $${T}_{j}=\prod_{k=1}^{j-1}{C}_{k}\left(x\right)\left(j=2,3,\dots ,n\right)$$, $${T}_{1}=1$$.

#### Pythagorean Fuzzy Schweizer–Sklar Operations

The Schweizer–Sklar product and Schweizer–Sklar sum, which are special examples of the Archimedean t-norm and t-conorm, respectively, are involved in Schweizer-Sklar operations.

##### Definition 8

[40] Assume $x$ and $y$ are any two real numbers. Then the definitions of the Schweizer–Sklar t-norm and t-conorm are shown as follows:

7 $${T}_{\eta }\left(x,y\right)={\left({x}^{\eta }+{y}^{\eta }-1\right)}^{1/\eta },$$

8 $${T}_{\eta }^{*}\left(x,y\right)=1-{\left({\left(1-x\right)}^{\eta }+{\left(1-y\right)}^{\eta }-1\right)}^{1/\eta },$$

where $$\eta <0;x,y\in \left[0,1\right]$$.

The Schweizer–Sklar operational rules on PFNs using Schweizer–Sklar t-norm and t-conorm are introduced as the following:

##### Definition 9

[41] Let $\alpha =\langle {\mu }_{\alpha },{\nu }_{\alpha }\rangle$,${\alpha }_{i}=\langle {\mu }_{i},{\nu }_{i}\rangle \left(i=1,2\right)$ be three PFNs, and let $\eta <0,\gamma >0$. Then Schweizer–Sklar operations of the t-norm and t-conorm of PFNs are proposed as follows:

1. $${\alpha }_{1}\oplus {\alpha }_{2}=\left\langle \sqrt{1-{\left[{\left(1-{\mu }_{1}^{2}\right)}^{\eta }+{\left(1-{\mu }_{2}^{2}\right)}^{\eta }-1\right]}^{1/\eta }},\sqrt{{\left[{\nu }_{1}^{2\eta }+{{\nu }_{2}^{2}}^{\eta }-1\right]}^{1/\eta }}\right\rangle$$
2. $${\alpha }_{1}\otimes {\alpha }_{2}=\left\langle \sqrt{{\left[{\mu }_{1}^{2\eta }+{\mu }_{2}^{2\eta }-1\right]}^{1/\eta }},\sqrt{1-{\left[{\left(1-{\nu }_{1}^{2}\right)}^{\eta }+{\left(1-{\nu }_{2}^{2}\right)}^{\eta }-1\right]}^{1/\eta }}\right\rangle $$
3. $$\gamma \alpha =\left\langle \sqrt{1-{\left[\gamma {\left(1-{\mu }_{\alpha }^{2}\right)}^{\eta }-\left(\gamma -1\right)\right]}^{1/\eta }}, \sqrt{{\left[{\nu }_{\alpha }^{2\eta }-\left(\gamma -1\right)\right]}^{1/\eta }}\right\rangle $$.
4. $${\alpha }^{\gamma }=\left\langle \sqrt{{\left[\gamma {\mu }_{\alpha }^{2\eta }-\left(\gamma -1\right)\right]}^{1/\eta }},\sqrt{1-{\left[\gamma {\left(1-{\nu }_{\alpha }^{2}\right)}^{\eta }-\left(\gamma -1\right)\right]}^{1/\eta }}\right\rangle .$$

### Pythagorean Fuzzy Schweizer–Sklar Prioritized Weighted Average Operator

In this subsection, according to the operational rules of PFNs with respect to Schweizer–Sklar operations in Definition 9 and the advantages of the PA operator in Definition 7, the PFSSPWA operator is established, and its enviable properties are discussed.

#### Definition 10

Let ${\alpha }_{i}=\langle {\mu }_{i},{\nu }_{i}\rangle \left(i=1,2,\dots ,n\right)$ be a set of PFNs, then the PFSSPWA operator is a function ${\text{PFSSPWA}}:{\text{PF}}{N}^{n}\to {\text{PFN}}$ such that:

9 $${\text{PFSSPWA}}\left({\alpha }_{1},{\alpha }_{2},\ldots ,{\alpha }_{n}\right)=\stackrel{n}{\underset{i=1}{\oplus}}\frac{{T}_{i}}{\sum_{i=1}^{n}{T}_{i}}{\alpha }_{i}=\frac{{T}_{1}}{\sum_{i=1}^{n}{T}_{i}}{\alpha }_{1}\oplus \frac{{T}_{2}}{\sum_{i=1}^{n}{T}_{i}}{\alpha }_{2}\oplus \cdots \oplus \frac{{T}_{n}}{\sum_{i=1}^{n}{T}_{i}}{\alpha }_{n},$$

where ${T}_{1}=1$ and ${T}_{i}=\prod_{k=1}^{i-1}S\left({\alpha }_{k}\right),\left(i=2,3,\ldots ,n\right)$. Here, $S\left({\alpha }_{k}\right)$ expresses the score value of PFNs.

We obtain the following theorem that follows the Schweizer–Sklar operations on PFNs.

#### Theorem 1

*Suppose* ${\alpha }_{i}=\langle {\mu }_{i},{\nu }_{i}\rangle \left(i=1,2,\dots ,n\right)$* is a set of PFNs and* $\eta <0$,* then the value aggregated by the proposed PFSSPWA operator is also a PFE and is specified by*:

10 $$\begin{aligned}{\text{PFSSPWA}}\left({\alpha }_{1},{\alpha }_{2},\ldots ,{\alpha }_{n}\right)=\left\langle \sqrt{1-{\left[\sum_{i=1}^{n}\frac{{T}_{i}}{{\sum }_{i=1}^{n}{T}_{i}}{\left(1-{\mu }_{i}^{2}\right)}^{\eta }\right]}^{1/\eta }} \right.,\\ \left. \sqrt{{\left(\sum_{i=1}^{n}\frac{{T}_{i}}{{\sum }_{i=1}^{n}{T}_{i}}{\nu }_{i}^{2\eta }\right)}^{1/\eta }}\right\rangle. \end{aligned}$$

#### ***Proof***

From the mathematical induction, Eq. (10) can be proved as follows:

1. If $n=2$, we have
  $$\begin{aligned}\frac{{T}_{1}}{{\sum }_{i=1}^{2}{T}_{i}}{\alpha }_{1}=\left\langle \sqrt{1-{\left[\frac{{T}_{1}}{{\sum }_{i=1}^{2}{T}_{i}}{\left(1-{\mu }_{1}^{2}\right)}^{\eta }-\left(\frac{{T}_{1}}{{\sum }_{i=1}^{2}{T}_{i}}-1\right)\right]}^{1/\eta }} \right. , \\ \left. \sqrt{{\left[\frac{{T}_{1}}{{\sum }_{i=1}^{2}{T}_{i}}{\nu }_{1}^{2\eta }-\left(\frac{{T}_{1}}{{\sum }_{i=1}^{2}{T}_{i}}-1\right)\right]}^{1/\eta }}\right\rangle \end{aligned} $$
  $$\begin{aligned}\frac{{T}_{2}}{{\sum }_{i=1}^{2}{T}_{i}}{\alpha }_{2}=\left\langle \sqrt{1-{\left[\frac{{T}_{2}}{{\sum }_{i=1}^{2}{T}_{i}}{\left(1-{\mu }_{2}^{2}\right)}^{\eta }-\left(\frac{{T}_{2}}{{\sum }_{i=1}^{2}{T}_{i}}-1\right)\right]}^{1/\eta }} \right., \\ \left.  \sqrt{{\left[\frac{{T}_{2}}{{\sum }_{i=1}^{2}{T}_{i}}{\nu }_{2}^{2\eta }-\left(\frac{{T}_{2}}{{\sum }_{i=1}^{2}{T}_{i}}-1\right)\right]}^{1/\eta }}\right\rangle \end{aligned}$$

Then

$$\frac{{T}_{1}}{\sum_{i=1}^{n}{T}_{i}}{\alpha }_{1}\oplus \frac{{T}_{2}}{\sum_{i=1}^{n}{T}_{i}}{\alpha }_{2}=\left\langle \sqrt{1-{\left[\frac{{T}_{1}}{{\sum }_{i=1}^{2}{T}_{i}}{\left(1-{\mu }_{1}^{2}\right)}^{\eta }-\left(\frac{{T}_{1}}{{\sum }_{i=1}^{2}{T}_{i}}-1\right)+\frac{{T}_{2}}{{\sum }_{i=1}^{2}{T}_{i}}{\left(1-{\mu }_{2}^{2}\right)}^{\eta }-\left(\frac{{T}_{2}}{{\sum }_{i=1}^{2}{T}_{i}}-1\right)-1\right]}^{1/\eta }}, \sqrt{{\left[\frac{{T}_{1}}{{\sum }_{i=1}^{2}{T}_{i}}{\nu }_{1}^{2\eta }-\left(\frac{{T}_{1}}{{\sum }_{i=1}^{2}{T}_{i}}-1\right)+\frac{{T}_{2}}{{\sum }_{i=1}^{2}{T}_{i}}{\nu }_{2}^{2\eta }-\left(\frac{{T}_{2}}{{\sum }_{i=1}^{2}{T}_{i}}-1\right)\right]}^{1/\eta }}\right\rangle =\Bigg\langle \sqrt{1-{\left[\sum_{i=1}^{2}\frac{{T}_{i}}{{\sum }_{i=1}^{2}{T}_{i}}{\left(1-{\mu }_{i}^{2}\right)}^{\eta }-\left(\sum_{i=1}^{2}\frac{{T}_{i}}{{\sum }_{i=1}^{2}{T}_{i}}-1\right)\right]}^{1/\eta }},\sqrt{{\left[\sum_{i=1}^{2}\frac{{T}_{i}}{{\sum }_{i=1}^{n}{T}_{i}}{\nu }_{i}^{2\eta }-\left(\sum_{i=1}^{2}\frac{{T}_{i}}{{\sum }_{i=1}^{2}{T}_{i}}-1\right)\right]}^{1/\eta }}=\bigg\langle \sqrt{1-{\left[\sum_{i=1}^{2}\frac{{T}_{i}}{{\sum }_{i=1}^{2}{T}_{i}}{\left(1-{\mu }_{i}^{2}\right)}^{\eta }\right]}^{1/\eta }},\sqrt{{\left[\sum_{i=1}^{2}\frac{{T}_{i}}{{\sum }_{i=1}^{n}{T}_{i}}{\nu }_{i}^{2\eta }\right]}^{1/\eta }}$$

Thus, when $n=2$, Eq. (10) is correct.

(2) Suppose $n=m$, then Eq. (10) is correct, and we have
  $$\mathrm{PFSSPWA}\left({\alpha }_{1},{\alpha }_{2},\dots ,{\alpha }_{m}\right)=\left\langle \sqrt{1-{\left[\sum_{i=1}^{m}\frac{{T}_{i}}{{\sum }_{i=1}^{m}{T}_{i}}{\left(1-{\mu }_{i}^{2}\right)}^{\eta }-\left(\sum_{i=1}^{m}\frac{{T}_{i}}{{\sum }_{i=1}^{m}{T}_{i}}-1\right)\right]}^{1/\eta }},\sqrt{{\left(\sum_{i=1}^{m}\frac{{T}_{i}}{{\sum }_{i=1}^{m}{T}_{i}}{\nu }_{i}^{2\eta }-\left(\sum_{i=1}^{m}\frac{{T}_{i}}{{\sum }_{i=1}^{m}{T}_{i}}-1\right)\right)}^{1/\eta }}\right\rangle =\left\langle \sqrt{1-{\left[\sum_{i=1}^{m}\frac{{T}_{i}}{{\sum }_{i=1}^{m}{T}_{i}}{\left(1-{\mu }_{i}^{2}\right)}^{\eta }\right]}^{1/\eta }},\sqrt{{\left(\sum_{i=1}^{m}\frac{{T}_{i}}{{\sum }_{i=1}^{m}{T}_{i}}{\nu }_{i}^{2\eta }\right)}^{1/\eta }}\right\rangle $$
(3) If $n=m+1$, we have
  $$\mathrm{PFSSPWA}\left({\alpha }_{1},{\alpha }_{2},\dots ,{\alpha }_{m+1}\right)=\mathrm{PFSSPWA}\left({\alpha }_{1},{\alpha }_{2},\dots ,{\alpha }_{m}\right)\oplus \frac{{T}_{m+1}}{{\sum }_{i=1}^{m+}{T}_{i}}{\alpha }_{m+1}\bigg\langle \sqrt{1-{\left[\sum_{i=1}^{m}\frac{{T}_{i}}{{\sum }_{i=1}^{m+1}{T}_{i}}{\left(1-{\mu }_{i}^{2}\right)}^{\eta }-\left(\sum_{i=1}^{m}\frac{{T}_{i}}{{\sum }_{i=1}^{m+1}{T}_{i}}-1\right)+\frac{{T}_{m+1}}{{\sum }_{i=1}^{n}{T}_{i}}{\left(1-{\mu }_{m+1}^{2}\right)}^{\eta }-\left(\frac{{T}_{m+1}}{{\sum }_{i=1}^{n}{T}_{i}}-1\right)-1\right]}^{1/\eta }},$$
  $$ \left. {\sqrt {\left( {\sum\limits_{{i = 1}}^{m} {\frac{{T_{i} }}{{\sum _{{i = 1}}^{m} T_{i} }}} \nu _{i}^{{2\eta }}  - \left( {\sum\limits_{{i = 1}}^{m} {\frac{{T_{i} }}{{\sum _{{i = 1}}^{m} T_{i} }}}  - 1} \right) + \frac{{T_{{m + 1}} }}{{\sum _{{i = 1}}^{n} T_{i} }}\nu _{{m + 1}}^{{2\eta }}  - \left( {\frac{{T_{{m + 1}} }}{{\sum _{{i = 1}}^{n} T_{i} }} - 1} \right)} \right)^{{1/\eta }} } } \right\rangle  = \left\langle {\sqrt {1 - \left[ {\sum\limits_{{i = 1}}^{{m + 1}} {\frac{{T_{i} }}{{\sum _{{i = 1}}^{{m + 1}} T_{i} }}} \left( {1 - \mu _{i}^{2} } \right)^{\eta }  - \left( {\sum\limits_{{i = 1}}^{{m + 1}} {\frac{{T_{i} }}{{\sum _{{i = 1}}^{{m + 1}} T_{i} }}}  - 1} \right)} \right]^{{1/\eta }} } ,\sqrt {\left( {\sum\limits_{{i = 1}}^{{m + 1}} {\frac{{T_{i} }}{{\sum _{{i = 1}}^{{m + 1}} T_{i} }}} \nu _{i}^{{2\eta }}  - \left( {\sum\limits_{{i = 1}}^{{m + 1}} {\frac{{T_{i} }}{{\sum _{{i = 1}}^{{m + 1}} T_{i} }}}  - 1} \right)} \right)^{{1/\eta }} } } \right\rangle  = \left\langle {\sqrt {1 - \left[ {\sum\limits_{{i = 1}}^{{m + 1}} {\frac{{T_{i} }}{{\sum _{{i = 1}}^{{m + 1}} T_{i} }}} \left( {1 - \mu _{i}^{2} } \right)^{\eta } } \right]^{{1/\eta }} } ,\sqrt {\left( {\sum\limits_{{i = 1}}^{{m + 1}} {\frac{{T_{i} }}{{\sum _{{i = 1}}^{{m + 1}} T_{i} }}} \nu _{i}^{{2\eta }} } \right)^{{1/\eta }} } } \right\rangle  $$

Thus, when $n=m+1$, Eq. (10) is also correct. Hence, Theorem 1 is proved.

#### Theorem 2

(Idempotency) *Assume* ${\alpha }_{i}=\langle {\mu }_{i},{\nu }_{i}\rangle \left(i=1,2,\dots ,n\right)$ *is a set of PFNs*; *if* ${\alpha }_{i}=\alpha =\langle \mu ,\nu \rangle ,\left(i=1,2,\dots ,n\right)$, *then* ${\text{PFSSPWA}}=\left({\alpha }_{1},{\alpha }_{2},\dots ,{\alpha }_{n}\right)=\alpha$.

#### ***Proof***

$$\begin{array}{l}{\text{PFSSPWA}}\left({\alpha }_{1},{\alpha }_{2},\dots ,{\alpha }_{n}\right)=\left\langle \sqrt{1-{\left(\sum_{i=1}^{n}\frac{{T}_{n}}{{\sum }_{i=1}^{n}{T}_{i}}{\left(1-{\mu }_{i}^{2}\right)}^{\eta } \right)}^{\frac{1}{\eta }}},\sqrt{{\left(\sum_{i=1}^{n}\frac{{T}_{i}}{{\sum }_{i=1}^{m}{T}_{i}}{\nu }_{i}^{2\eta }\right)}^{1/\eta }}\right\rangle \\ =\left\langle \sqrt{1-{\left(\sum_{i=1}^{n}\frac{{T}_{n}}{{\sum }_{i=1}^{n}{T}_{i}}{\left(1-{\mu }^{2}\right)}^{\eta } \right)}^{\frac{1}{\eta }}},\sqrt{{\left(\sum_{i=1}^{n}\frac{{T}_{i}}{{\sum }_{i=1}^{m}{T}_{i}}{\nu }^{2\eta }\right)}^{1/\eta }}\right\rangle =\langle \mu ,\nu \rangle =\alpha \end{array}$$

Then, the proof of Theorem 2 is completed.

#### Theorem 3

(Monotonicity) *Assume* ${\alpha }_{i}=\langle {\mu }_{{\alpha }_{i}},{\nu }_{{\alpha }_{i}}\rangle$ *and* ${\beta }_{i}=\langle {\mu }_{{\beta }_{i}},{\nu }_{{\beta }_{i}}\rangle$, $i=\left(1,2,\dots ,n\right)$ *as two sets of PFNs; if* ${\mu }_{{\alpha }_{i}} \leqslant  {\mu }_{{\beta }_{i}},{\nu }_{{\alpha }_{i}} \leqslant  {\nu }_{{\beta }_{i}},\forall i\in \left\{1,2,\dots ,n\right\}$, *then* $\mathrm{PFSSPWA}\left({\alpha }_{1},{\alpha }_{2},\dots ,{\alpha }_{n}\right) \leqslant  \mathrm{PFSSPWA}\left({\beta }_{1},{\beta }_{2},\dots ,{\beta }_{n}\right)$.

#### ***Proof***

Since $${\mu }_{{\alpha }_{i}} \leqslant  {\mu }_{{\beta }_{i}},\forall i\in \left\{1,2,\ldots ,n\right\}$$,

$$\frac{{T}_{n}}{{\sum }_{i=1}^{n}{T}_{i}}{\left(1-{\mu }_{{\alpha }_{i}}^{2}\right)}^{\eta } \leqslant  \frac{{T}_{n}}{{\sum }_{i=1}^{n}{T}_{i}}{\left(1-{\mu }_{{\beta }_{i}}^{2}\right)}^{\eta }\Rightarrow \sqrt{1-{\left(\sum_{i=1}^{n}\frac{{T}_{n}}{{\sum }_{i=1}^{n}{T}_{i}}{\left(1-{\mu }_{{\alpha }_{i}}^{2}\right)}^{\eta } \right)}^{\frac{1}{\eta }}} \leqslant  \sqrt{1-{\left(\sum_{i=1}^{n}\frac{{T}_{n}}{{\sum }_{i=1}^{n}{T}_{i}}{\left(1-{\mu }_{{\beta }_{i}}^{2}\right)}^{\eta } \right)}^{\frac{1}{\eta }}}$$

Similarly, if ${\nu }_{{\alpha }_{i}} \leqslant  {\nu }_{{\beta }_{i}},\forall i\in \left\{1,2,\dots ,n\right\}$, the following results can be obtained.

$$\sqrt{{\left(\sum_{i=1}^{n}\frac{{T}_{i}}{{\sum }_{i=1}^{m}{T}_{i}}{\nu }_{{\alpha }_{i}}^{2\eta }\right)}^{1/\eta }} \leqslant  \sqrt{{\left(\sum_{i=1}^{n}\frac{{T}_{i}}{{\sum }_{i=1}^{m}{T}_{i}}{\nu }_{{\beta }_{i}}^{2\eta }\right)}^{1/\eta }}$$

Thus, Theorem 3 can be obtained.

#### Theorem 4

(Boundedness) *Assume* ${\alpha }_{i}=\langle {\mu }_{i},{\nu }_{i}\rangle \left(i=1,2,\dots ,n\right)$ *is a set of PFNs*;* if* ${\alpha }_{\mathrm{min}}=\langle \underset{i}{\mathrm{min}}\left({\mu }_{i}\right),\underset{i}{\mathrm{max}}\left({\nu }_{i}\right)\rangle$,${\alpha }_{\mathrm{max}}=\langle \underset{i}{\mathrm{max}}\left({\mu }_{i}\right),\underset{i}{\mathrm{min}}\left({\nu }_{i}\right)\rangle$, *then* ${\alpha }_{\mathrm{min}} \leqslant  \mathrm{PFSSPWA}\left({\alpha }_{1},{\alpha }_{2},\dots ,{\alpha }_{n}\right) \leqslant  {\alpha }_{\mathrm{max}}$.

#### Proof

Since $\underset{i}{\mathrm{min}}\left({\mu }_{i}\right) \leqslant  {\mu }_{i} \leqslant  \underset{i}{\mathrm{max}}\left({\mu }_{i}\right)$ and $\underset{i}{\mathrm{min}}\left({\nu }_{i}\right) \leqslant  {\nu }_{i} \leqslant  \underset{i}{\mathrm{max}}\left({\nu }_{i}\right)$, for $\forall i\in \left\{1,2,\ldots ,n\right\}$, then from Theorem 3, we can get

$$\mathrm{PFSSPWA}\left({\alpha }_{\mathrm{min}},{\alpha }_{\mathrm{min}},\dots ,{\alpha }_{\mathrm{min}}\right) \leqslant  \mathrm{PFSSPWA}\left({\alpha }_{1},{\alpha }_{2},\dots ,{\alpha }_{n}\right)\Rightarrow {\alpha }_{\mathrm{min}} \leqslant  \mathrm{PFSSPWA}\left({\alpha }_{1},{\alpha }_{2},\dots ,{\alpha }_{n}\right)$$

Similarly,

$$\mathrm{PFSSPWA}\left({\alpha }_{\mathrm{max}},{\alpha }_{\mathrm{max}},\dots ,{\alpha }_{\mathrm{max}}\right) \geqslant \mathrm{PFSSPWA}\left({\alpha }_{1},{\alpha }_{2},\dots ,{\alpha }_{n}\right)\Rightarrow {\alpha }_{\mathrm{max}} \geqslant \mathrm{PFSSPWA}\left({\alpha }_{1},{\alpha }_{2},\dots ,{\alpha }_{n}\right)$$

Thus, Theorem 4 is true.

### Pythagorean Fuzzy Schweizer–Sklar Prioritized weighted Geometric Operator

In this subsection, the PFSSPWG operator is proposed and some of its desirable properties are investigated in detail.

#### Definition 11

*Let* ${\alpha }_{i}=\langle {\mu }_{i},{\nu }_{i}\rangle \left(i=1,2,\ldots ,n\right)$ *be a set of PFNs, then the PFSSPWG operator is a function* $\mathrm{PFSSPWG}:{\mathrm{PFN}}^{n}\to \mathrm{PFN}$ such that:

11 $$\mathrm{PFSSPWG}\left({\alpha }_{1},{\alpha }_{2},\dots ,{\alpha }_{n}\right)=\stackrel{n}{\underset{i=1}{\otimes}}{{\alpha }_{i}}^{\frac{{T}_{i}}{\sum_{i=1}^{n}{T}_{i}}}={{\alpha }_{1}}^{\frac{{T}_{1}}{\sum_{i=1}^{n}{T}_{i}}}\otimes {{\alpha }_{2}}^{\frac{{T}_{2}}{\sum_{i=1}^{n}{T}_{i}}}\otimes \cdots \otimes {{\alpha }_{n}}^{\frac{{T}_{n}}{\sum_{i=1}^{n}{T}_{i}}},$$

where ${T}_{1}=1$ and ${T}_{i}=\prod_{k=1}^{i-1}S\left({\alpha }_{k}\right),\left(i=2,3,\ldots ,n\right)$. Here, $S\left({\alpha }_{k}\right)$ expresses the score value of PFNs.

#### Theorem 5

*Suppose* ${\alpha }_{i}=\langle {\mu }_{i},{\nu }_{i}\rangle \left(i=1,2,\dots ,n\right)$ *is a set of PFNs and* $\eta <0$, *then the value aggregated by the proposed PFSSPWG operator is also a PFN and is specified by*:

12 $$\mathrm{PFSSPWG}\left({\alpha }_{1},{\alpha }_{2},\dots ,{\alpha }_{n}\right)=\left\langle \sqrt{{\left(\sum_{i=1}^{n}\frac{{T}_{i}}{{\sum }_{i=1}^{n}{T}_{i}}{\mu }_{i}^{2\eta }\right)}^{1/\eta }},\sqrt{1-{\left[\sum_{i=1}^{n}\frac{{T}_{i}}{{\sum }_{i=1}^{n}{T}_{i}}{\left(1-{\nu }_{i}^{2}\right)}^{\eta }\right]}^{1/\eta }}\right\rangle .$$

The proof of this theorem is similar to the proof of Theorem 1.

Similar to Theorems 2–4, it can be easily proven that the PFSSPWG operator has the following properties:

#### Theorem 6

(Idempotency) *Assume* ${\alpha }_{i}=\langle {\mu }_{i},{\nu }_{i}\rangle \left(i=1,2,\dots ,n\right)$ *is a set of PFNs; if* ${\alpha }_{i}=\alpha =\langle \mu ,\nu \rangle ,\left(i=1,2,\dots ,n\right)$, *then* $PFSSPWG=\left({\alpha }_{1},{\alpha }_{2},\dots ,{\alpha }_{n}\right)=\alpha$.

#### Theorem 7

(Monotonicity) *Assume* ${\alpha }_{i}=\langle {\mu }_{{\alpha }_{i}},{\nu }_{{\alpha }_{i}}\rangle$ *and* ${\beta }_{i}=\langle {\mu }_{{\beta }_{i}},{\nu }_{{\beta }_{i}}\rangle$, $i=\left(1,2,\dots ,n\right)$ *as two sets of PFNs; if* ${\mu }_{{\alpha }_{i}} \leqslant  {\mu }_{{\beta }_{i}},{\nu }_{{\alpha }_{i}} \leqslant  {\nu }_{{\beta }_{i}},\forall i\in \left\{1,2,\dots ,n\right\}$, *then* $PFSSPWG\left({\alpha }_{1},{\alpha }_{2},\dots ,{\alpha }_{n}\right) \leqslant  PFSSPWG\left({\beta }_{1},{\beta }_{2},\dots ,{\beta }_{n}\right)$.

#### Theorem 8

(Boundedness) *Assume* ${\alpha }_{i}=\langle {\mu }_{i},{\nu }_{i}\rangle \left(i=1,2,\ldots ,n\right)$ *is a set of PFNs; if* ${\alpha }_{\mathrm{min}}=\langle \underset{i}{\mathrm{min}}\left({\mu }_{i}\right),\underset{i}{\mathrm{max}}\left({\nu }_{i}\right)\rangle$,${\alpha }_{\mathrm{max}}=\langle \underset{i}{\mathrm{max}}\left({\mu }_{i}\right),\underset{i}{\mathrm{min}}\left({\nu }_{i}\right)\rangle$, *then* ${\alpha }_{\mathrm{min}} \leqslant  PFSSPWG\left({\alpha }_{1},{\alpha }_{2},\dots ,{\alpha }_{n}\right) \leqslant  {\alpha }_{\mathrm{max}}$.

### A New MULTIMOORA Method Based on Pythagorean Fuzzy Schweizer–Sklar Prioritized Aggregation Operators

**Fig. 1:**
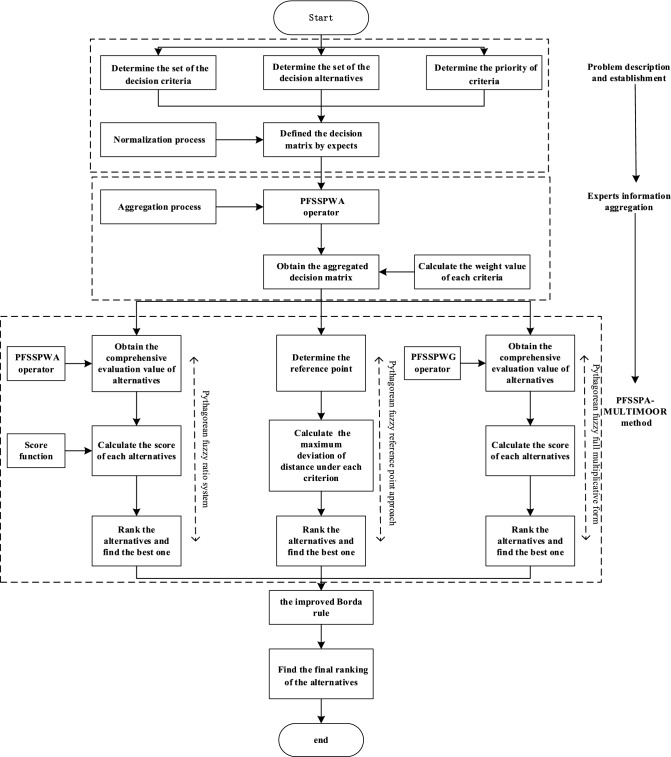
Flow chart for the PFSSPA-MULTIMOORA method

This subsection utilizes the proposed Pythagorean fuzzy Schweizer–Sklar prioritized aggregation operators and the existing classical MULTIMOORA method to develop a new MULTIMOORA method (named PFSSPA-MULTIMOORA) for MCDM in Pythagorean fuzzy environments. The following assumptions or notations are used to illustrate the considered problem. Let $A=\left({a}_{1},{a}_{2},\dots ,{a}_{m}\right)$ represent a set of alternatives and $C=\left({c}_{1},{c}_{2},\dots ,{c}_{n}\right)$ represent a set of criteria that satisfy the prioritization condition: ${c}_{1}\succ {c}_{2}\succ {c}_{3}\succ \cdots \succ {c}_{n}$, which indicates that criterion ${c}_{i}$ has a higher significance than ${c}_{j}$, for $\forall i<j$. Suppose that ${D}^{t}={\left({x}_{ij}^{t}\right)}_{m\times n}={\langle {\mu }_{ij},{\nu }_{ij}\rangle }_{m\times n}$ is the Pythagorean fuzzy decision matrix provided by experts $t=\left(1,2,\ldots ,p\right)$ to evaluate the degrees of the alternative ${x}_{i}\left(i=1,2,\dots ,m\right)$ which satisfy the criterion ${C}_{j}\left(j=1,2,\dots ,n\right)$. The prioritization of experts satisfies ${t}_{1}\succ {t}_{2}\succ \cdots \succ {t}_{p}$. Then the decision-making process for the PFSSPA-MULTIMOORA method is shown in Fig. 1.

As shown in Fig. 1, the method involves the following steps.

Step 1: The decision matrix is constructed by experts in Pythagorean fuzzy environments.

In the MCDM process, the criteria of alternatives can be determined by experts, and they need to give corresponding evaluation values. The corresponding decision matrix is denoted as ${D}^{t}={\left({x}_{ij}^{t}\right)}_{m\times n}={\langle {\mu }_{ij},{\nu }_{ij}\rangle }_{m\times n}$.

Step 2: Normalize the data.

Generally, there are two forms of criteria in the MCDM problem, including the benefit type and the cost type. To eliminate the influence caused by different types, the criteria should be transformed into the same type. Then the data normalization of each ${x}_{ij}$ can be computed by Eq. (13).

13 $${x}_{ij}^{\mathrm{^{\prime}}}=\left\{\begin{array}{ll}{x}_{ij}=\left\langle {\mu }_{ij},{\nu }_{ij}\right\rangle & \mathrm{if }\, c\, \mathrm{is}\, \mathrm{the}\, \mathrm{benifit} \,\mathrm{type}\\ {x}_{ij}^{c}=\left\langle {\nu }_{ij},{\mu }_{ij}\right\rangle & \mathrm{if }\, c \, \mathrm{is}\,  \mathrm{the \,cost} \, \mathrm{type},\end{array}\right.$$

where ${x}_{ij}^{c}$ denotes the complement of ${x}_{ij}$.

Step 3: Aggregate the individual decision opinions to a comprehensive decision matrix.

In this step, we can utilize the PFSSPWA to create the aggregated decision matrix ${D}^{*}={\left({x}_{ij}^{*}\right)}_{m\times n}$, where the weight vector ${V}_{t}=\left({v}_{1},{v}_{2},\dots ,p\right)$ holds for different experts. Then the aggregated evaluation values of all experts can be determined as:

14 $${x}_{ij}^{*}=\mathrm{PFSSPWA}\left({x}_{ij}^{1},{x}_{ij}^{2},\ldots ,{x}_{ij}^{p}\right)=\left\langle \sqrt{1-{\left[\sum_{i=1}^{p}{\upsilon }_{i}{\left(1-{\mu }_{ij}^{2}\right)}^{\eta }\right]}^{1/\eta }},\sqrt{{\left(\sum_{i=1}^{p}{\upsilon }_{i}{\nu }_{ij}^{2\eta }\right)}^{1/\eta }}\right\rangle ,$$

where $\sum_{i=1}^{p}{v}_{i}=1$, the value of ${v}_{1}>{v}_{2}>\cdots >{v}_{p}$ represents the prioritization of experts, and the weight value is given in some cases.

Step 4: The Pythagorean fuzzy ratio system (PF-RS) involves the following three steps:

(1) Aggregate the Pythagorean fuzzy values of alternatives into collective values using the proposed Pythagorean fuzzy Schweizer–Sklar prioritized weighted average operator, i.e.,

15 $${y}_{i}={\text{PFSSPWA}}\left({x}_{i1}^{*},{x}_{i2}^{*},\ldots ,{x}_{in}^{*}\right)=\left\langle \sqrt{1-{\left[\sum_{i=1}^{m}\frac{{T}_{ij}}{{\sum }_{j=1}^{n}{T}_{ij}}{\left(1-{\mu }_{ij}^{2}\right)}^{\eta }\right]}^{1/\eta }},\sqrt{{\left(\sum_{i=1}^{m}\frac{{T}_{ij}}{{\sum }_{j=1}^{n}{T}_{ij}}{\nu }_{ij}^{2\eta }\right)}^{1/\eta }}\right\rangle ,$$

where $${T}_{ij}=\prod_{k=1}^{j-1}S\left({x}_{ik}\right)$$ and $${T}_{i1}=1\left(i=1,2,\dots , m; \, j=2,3,\dots ,n\right)$$.

(2) Equation. (3) is utilized to calculate the score value ${S}_{1}\left({y}_{i}\right)$ of each alternative.

(3) Rank the alternatives. The higher the value of ${S}_{1}\left({y}_{i}\right)$, the better the alternatives ${a}_{i}$, i.e.,

16 $${a}_{RS}^{*}=\left\{{a}_{i}\left|\underset{i}{\mathrm{max}}{S}_{1}\left({y}_{i}\right)\right.\right\}$$

Step 5: Pythagorean fuzzy reference point (PF-RP) approach. The main idea of this method is to find a suitable reference point and calculate the distance of the alternatives to the reference point. It involves the following three steps:

(1) In this step, the Pythagorean fuzzy reference point $f=\left({f}_{1},{f}_{2}.\cdots ,{f}_{n}\right)$ can be calculated by Eq. (17)

17 $${f}_{j}=\left\{\begin{array}{ll}\left\langle \underset{i}{\mathrm{max}}{\mu }_{ij},\underset{i}{\mathrm{min}}{\nu }_{ij}\right\rangle & \mathrm{if} \, c \, \mathrm{is} \, \mathrm{the} \, \mathrm{benifit} \, \mathrm{type}\\ \left\langle \underset{i}{\mathrm{min}}{\mu }_{ij},\underset{i}{\mathrm{max}}{\nu }_{ij}\right\rangle & \mathrm{if} \, c \, \text{is the cost type}.\end{array}\right.$$

(2) Combined with the distance measure Eq. (5) of Pythagorean fuzzy numbers, calculate the normalized distance from the comprehensive evaluation value of alternatives to the reference point, i.e.,

18 $${S}_{2}\left({y}_{i}\right)=d\left({x}_{{}_{ij}}^{*},{f}_{j}\right)=\frac{1}{2}\left(\left|{\mu }_{{x}_{ij}^{*}}^{2}-{\mu }_{{f}_{j}}^{2}\right|+\left|{\nu }_{{x}_{ij}^{*}}^{2}-{\nu }_{{f}_{j}}^{2}\right|+\left|{\pi }_{{x}_{ij}^{*}}^{2}-{\pi }_{{f}_{j}}^{2}\right|\right).$$

(3) Rank the alternatives. The rank principle is that the lower the value of $d$ is, the better the alternatives ${a}_{i}$, i.e.,

19 $${a}_{RP}^{*}=\left\{{a}_{i}\left|\underset{i}{\mathrm{min}}{S}_{2}\left({y}_{i}\right)\right.\right\}.$$

Step 6: The Pythagorean fuzzy full multiplicative form (PF-FMF) mainly uses the product operator to assemble the evaluation matrix of each alternative. It also involves the following three steps:

(1) Aggregate the Pythagorean fuzzy values of alternatives into collective values using the proposed Pythagorean fuzzy Schweizer–Sklar prioritized weighted geometric operator, i.e.,

20 $$ \begin{aligned}   y_{i}  & = {\text{PFSSPWG}}\left( {x_{{i1}}^{*} ,x_{{i2}}^{*} , \ldots ,x_{{in}}^{*} } \right) \hfill \\    & = \left\langle {\sqrt {\left( {\sum\limits_{{i = 1}}^{m} {\frac{{T_{{ij}} }}{{\sum _{{j = 1}}^{n} T_{{ij}} }}} \mu _{{ij}}^{{2\eta }} } \right)^{{1/\eta }} } } \right., \hfill \\   & \quad \left. {\sqrt {1 - \left[ {\sum\limits_{{i = 1}}^{m} {\frac{{T_{{ij}} }}{{\sum _{{j = 1}}^{n} T_{{ij}} }}} \left( {1 - \nu _{{ij}}^{2} } \right)^{\eta } } \right]^{{1/\eta }} } } \right\rangle , \hfill \\  \end{aligned}  $$

where $${T}_{ij}=\prod_{k=1}^{j-1}S\left({x}_{ik}\right)$$ and $${T}_{i1}=1\left(i=1,2,\dots ,m;j=2,3,\dots ,n\right)$$.

(2) Equation. (3) is utilized to calculate the score value ${S}_{3}\left({y}_{i}\right)$ of each alternative.

(3) Rank the alternatives. The higher the value of ${S}_{3}\left({y}_{i}\right)$, the better the alternatives ${a}_{i}$, i.e.,

21 $${a}_{FMF}^{*}=\left\{{a}_{i}\left|\underset{i}{\mathrm{max}}{S}_{3}\left({y}_{i}\right)\right.\right\}.$$

Step 7: The improved Borda rule. Wu et al.[16] proposed the improved Borda rule to avoid the limitation of dominance theory. It considers the ranking values ${S}_{\zeta }\left({y}_{i}\right)\left(\zeta =1,2,3\right)$ of each alternative ${a}_{i}\left(i=1,2,\dots ,m\right)$ under three different subsystems (PF-RS, PF-RP, and PF-FMF). We can utilize the improved Borda rule to aggregate the above ranking results into the final ranking. The details are as follows:

Normalize the ranking values ${S}_{\zeta }\left({y}_{i}\right)$ of the three subsystems, i.e.,

22 $${S}_{\zeta }^{\mathrm{^{\prime}}}\left({y}_{i}\right)=\frac{{S}_{\zeta }\left({y}_{i}\right)}{\sqrt{{\sum }_{i=1}^{m}{\left({S}_{\zeta }^{\mathrm{^{\prime}}}\left({y}_{i}\right)\right)}^{2}}}.$$

The final ranking results of alternatives are computed as

23 $$ \begin{aligned}   \Psi _{i}  =  & S_{1}^{\prime } \left( {y_{i} } \right)\frac{{m - \rho _{1} \left( {y_{i} } \right) + 1}}{{m\left( {m + 1} \right)/2}} \\     & \quad  - S_{2}^{\prime } \left( {y_{i} } \right)\frac{{\rho _{2} \left( {y_{i} } \right)}}{{\frac{{m\left( {m + 1} \right)}}{2}}} + S_{3}^{\prime } \left( {y_{i} } \right)\frac{{m - \rho _{3} \left( {y_{i} } \right) + 1}}{{\frac{{m\left( {m + 1} \right)}}{2}}}, \\     & \quad \left( {i = 1,2, \ldots ,m} \right), \\  \end{aligned}  $$

where ${\rho }_{\zeta }\left({y}_{i}\right)$ denotes the ranking order of each alternative in three subsystems. Then the alternative with the higher final ranking result is better. Thus, the alternatives can be ranked in descending order of their final ranking results.

## Case Study

CO_2_ geological storage site selection is an important part of CCUS [5, 52, 56], which has received extensive attention and heated discussion in academic circles. This section treats CO_2_ geological storage site selection as an MCDM problem to demonstrate the applicability of the proposed method. The proposed method's results are then compared to those of other methods to validate the validity and benefits of our work.

### Application of the Proposed Method

Large-scale CO_2_ emissions have led to climate change. Reducing CO_2_ emissions and mitigating the impact of CO_2_ on climate change have become global goals. CCUS technology is an important way to reduce CO_2_ emissions. In general, CCUS comprises three main steps: CO_2_ capture from large point sources, CO_2_ transportation, and CO_2_ storage. China is the largest producer and consumer of coal in the world, and coal is the main source of energy. Coal accounts for 76% of China's primary energy consumption. It is predicted that this proportion will not change much over a long period of time. Therefore, CCUS has become one of the most hopeful technologies in China. CO_2_ storage site selection is an important part of CCUS project management; it is nearly the elementary procedure for the completion of CO_2_ geological storage [5]. Thus, on the premise of ensuring CO_2_ capture and transportation, it is necessary to form a complete decision method on how to select a suitable storage location.

Based on the literature, after several rounds of discussion by experts, there are five decision criteria $C=\left\{{c}_{1},{c}_{2},{c}_{3},{c}_{4},{c}_{5}\right\}$ for this evaluation. Moreover, the weights of the criteria are unknown and satisfy ${c}_{1}\succ {c}_{2}\succ {c}_{3}\succ {c}_{4}\succ {c}_{5}$.The details are as follows.

The environmental risk ${c}_{1}:$ Evaluation index is very important for the evaluation of CO_2_ geological storage. Any CO_2_ leakage during the several phases of the CCUS technology will have an ecological impact because of the characteristics of CO_2_ itself. The environmental risks during the stages of capture and transportation are often minor given the state of technology, and the main environmental risk is associated with the storage and use of CO_2_ in the earth's crust. Therefore, it is particularly important to conduct a scientific geological survey and identify related risk factors in the early stages of site selection.

Technical impact ${c}_{2}$: CO_2_ geological storage technology requires a high level of integration of various technologies, and it is necessary to promote the development of each link in an orderly and balanced manner. Since there is uncertainty and risk in geological exploration and geological storage, companies need to make a comprehensive assessment of stratigraphic structure, storage potential, storage risk, and other issues.

Investment costs ${c}_{3}$: The investment cost of CO_2_ geological storage mainly includes land development engineering expenses such as pre-engineering planning, design, and hydrogeological survey. It is an important indicator for decision makers to evaluate the feasibility of the project.

Social impact ${c}_{4}$: The social impact consists of the following two main aspects: (1) distance to residential sites. To ensure the safety of the project, the geological storage site was chosen as far away from residential areas as possible. (2) The construction and operation of the project will have a certain impact on the lives of nearby residents. The risk of CO_2_ leakage may reduce the acceptance of CCUS projects by nearby residents. Therefore, social impact is a necessary consideration for the construction of CCUS projects.

Policy support ${c}_{5}$: Since 2006, China has issued more than 20 national policies involving CCUS, establishing the importance of CCUS in addressing climate change, and actively promoting the CCUS technology and the construction of demonstration projects. However, special laws, regulations, and standard systems for CCUS have not yet been established. Therefore, it is very important to introduce clear government policies and establish special laws, regulations, and standards as soon as possible for the large-scale implementation of CCUS projects.

**Fig. 2:**
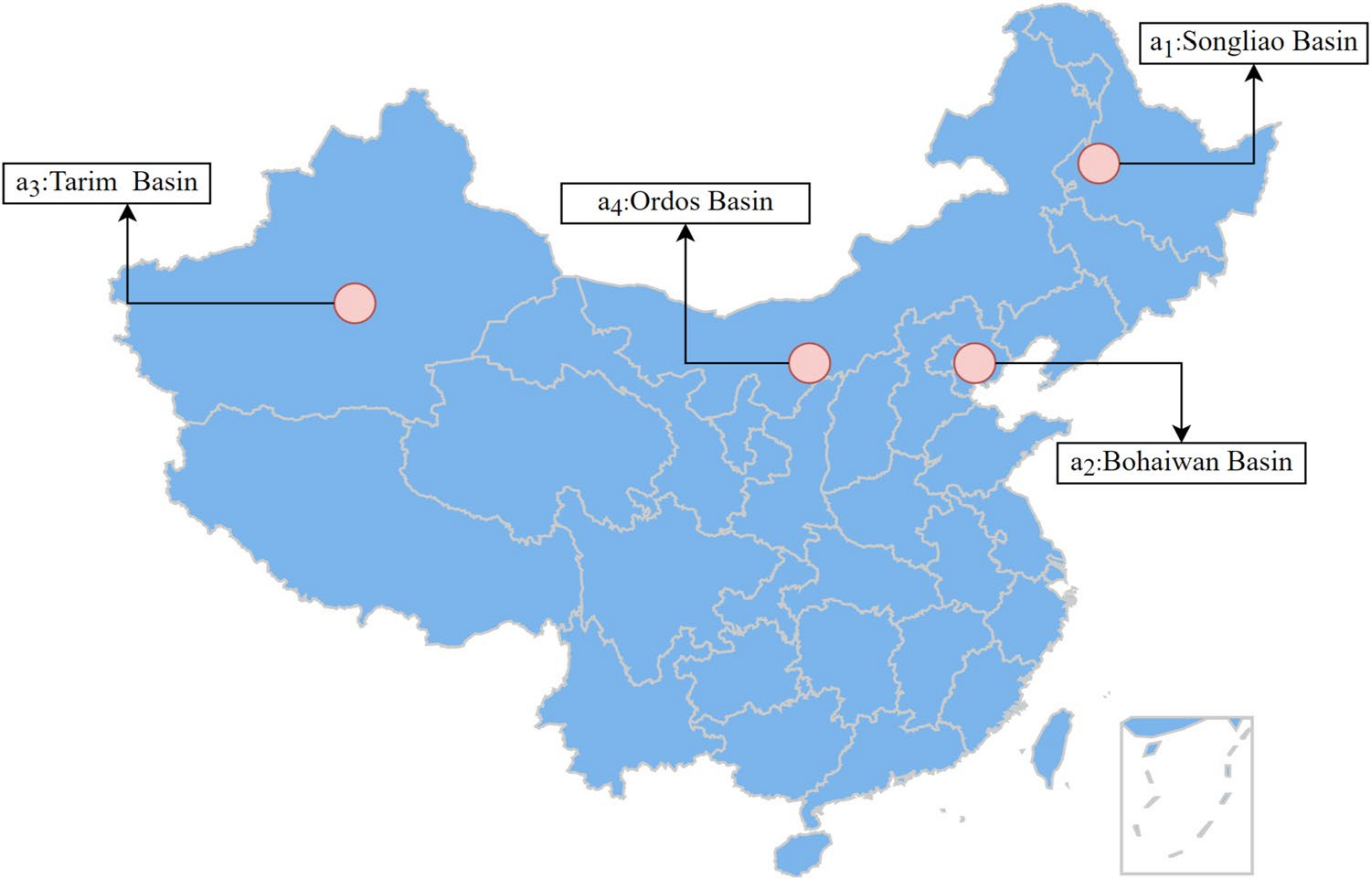
Distribution of the four sites

It is estimated that China can sequester 1.21 trillion to 4.13 trillion tons of carbon dioxide by geological storage alone, with huge carbon storage potential. In this study, after preliminary screening by the expert group, four storage sites were identified for further evaluation and selection. The specific information about the four candidate sites is as follows, and Fig. 2 shows the geographical location of the four sites.

The Songliao Basin (${a}_{1}$) is a large sedimentary basin in northeastern China, spanning four provinces: Heilongjiang, Jilin, Liaoning, and the Inner Mongolia Autonomous Region. There are many Middle Cenozoic oil-bearing basins around the basin, and there are 23 sedimentary basins with a deposition area larger than 500 km^2^. The storage potential is about 694.5 billion tons.

The Bohaiwan Basin (${a}_{2}$), an important oil- and gas-bearing basin in eastern China, is currently the basin with the highest total oil and gas production in China. The Bohaiwan Basin includes the cities of Beijing and Tianjin and parts of the four provinces of Hebei, Shandong, Henan, and Liaoning, as well as the waters of the Bohai Sea, covering an area of about 200,000 square kilometers. The basin includes seven major oil fields in Liaohe, North China, Dagang, Jidong, Shengli, Zhongyuan, and Bohai.

The Tarim Basin (${a}_{3}$) is located in the south of Xinjiang, China, between the Tianshan Mountains, Kunlun Mountains, and Alpine Mountains, and is the largest closed basin in China. The Tarim Basin is rich in oil and gas resources, especially natural gas reserves, which account for 1/4 of the country's land-based natural gas reserves. 18.4 billion tons of oil and gas resources are forecast, including 10.1 billion tons of oil and 8.3 trillion cubic meters of natural gas, making it the starting point for China's “West–East Gas Transmission”.

The Ordos Basin (${a}_{4}$) is located in the western part of the North China Plate, which is the second largest sedimentary basin and an important energy base in China. It is rich in oil and gas resources, featuring a wide distribution of oil and gas, many oil-bearing sections, and a large thickness of oil layers. The basin’s deep saline layer is widely distributed, with several reservoir-cover combinations suitable for CO_2_ geological storage, and the total CO_2_ storage potential is estimated to be in the tens of billions of tons, with broad storage prospects.

To ensure the scientificity and objectivity of this evaluation, experts from scientific research institutions, government departments, nonprofit organizations, and senior engineering technicians were invited to participate in the evaluation. At the beginning of the evaluation period, experts were under time pressure and had limited reference materials, and their own experience with related problems was also limited. Experts are willing to use Pythagorean fuzzy numbers to express their judgment on related problems. It is helpful to reflect uncertainty about the evaluation problem. Tables 4, 5, and 6 show the outcome of Pythagorean fuzzy group decision-making after standardization.

**Table 4:** Pythagorean fuzzy matrix given by expert 1

${D}^{1}\left({x}_{ij}\right)$	${c}_{1}$	${c}_{2}$	${c}_{3}$	${c}_{4}$	${c}_{5}$
${a}_{1}$	$\langle 0.7,0.3\rangle$	$\langle 0.9,0.2\rangle$	$\langle 0.6,0.3\rangle$	$\langle 0.4,0.7\rangle$	$\langle 0.6,0.3\rangle$
${a}_{2}$	$\langle 0.6,0.2\rangle$	$\langle 0.7,0.5\rangle$	$\langle 0.6,0.2\rangle$	$\langle 0.5,0.2\rangle$	$\langle 0.5,0.1\rangle$
${a}_{3}$	$\langle 0.5,0.3\rangle$	$\langle 0.7,0.1\rangle$	$\langle 0.7,0.4\rangle$	$\langle 0.7,0.2\rangle$	$\langle 0.9,0.3\rangle$
${a}_{4}$	$\langle 0.8,0.3\rangle$	$\langle 0.5,0.3\rangle$	$\langle 0.5,0.1\rangle$	$\langle 0.6,0.3\rangle$	$\langle 0.6,0.4\rangle$

**Table 5:** Pythagorean fuzzy matrix given by expert 2

${D}^{2}\left({x}_{ij}\right)$	${c}_{1}$	${c}_{2}$	${c}_{3}$	${c}_{4}$	${c}_{5}$
${a}_{1}$	$\langle 0.7,0.2\rangle$	$\langle 0.5,0.3\rangle$	$\langle 0.6,0.1\rangle$	$\langle 0.5,0.5\rangle$	$\langle 0.7,0.4\rangle$
${a}_{2}$	$\langle 0.5,0.3\rangle$	$\langle 0.7,0.5\rangle$	$\langle 0.7,0.4\rangle$	$\langle 0.7,0.5\rangle$	$\langle 0.6,0.1\rangle$
${a}_{3}$	$\langle 0.9,0.1\rangle$	$\langle 0.4,0.3\rangle$	$\langle 0.7,0.3\rangle$	$\langle 0.8,0.2\rangle$	$\langle 0.9,0.2\rangle$
${a}_{4}$	$\langle 0.6,0.5\rangle$	$\langle 0.8,0.1\rangle$	$\langle 0.5,0.3\rangle$	$\langle 0.5,0.3\rangle$	$\langle 0.5,0.2\rangle$

**Table 6:** Pythagorean fuzzy matrix given by expert 3

${D}^{3}\left({x}_{ij}\right)$	${c}_{1}$	${c}_{2}$	${c}_{3}$	${c}_{4}$	${c}_{5}$
${a}_{1}$	$\langle 0.5,0.3\rangle$	$\langle 0.6,0.5\rangle$	$\langle 0.6,0.2\rangle$	$\langle 0.5,0.5\rangle$	$\langle 0.6,0.4\rangle$
${a}_{2}$	$\langle 0.7,0.1\rangle$	$\langle 0.6,0.3\rangle$	$\langle 0.8,0.2\rangle$	$\langle 0.7,0.5\rangle$	$\langle 0.5,0.4\rangle$
${a}_{3}$	$\langle 0.6,0.3\rangle$	$\langle 0.8,0.2\rangle$	$\langle 0.7,0.4\rangle$	$\langle 0.6,0.2\rangle$	$\langle 0.7,0.6\rangle$
${a}_{4}$	$\langle 0.9,0.3\rangle$	$\langle 0.5,0.2\rangle$	$\langle 0.7,0.4\rangle$	$\langle 0.8,0.3\rangle$	$\langle 0.9,0.2\rangle$

The proposed PFSSPA-MULTIMOORA method is used to handle the above CO_2_ geological storage site selection problem as follows:

Step 1: The Pythagorean fuzzy matrices of each expert are shown in Tables 4, 5, and 6.

Step 2: Since the measurement scales of all the criteria are the same, there is no need to do so.

Step 3: Calculate the aggregated evaluation values of all experts using Eq. (14), and we let $\eta =-2$. The results are shown in Table 7, and the weight of each criterion is shown in Table 8.

**Table 7:** Expert group decision matrix

${D}^{*}\left({x}_{ij}\right)$	${c}_{1}$	${c}_{2}$	${c}_{3}$	${c}_{4}$	${c}_{5}$
${a}_{1}$	$\langle 0.68,0.24\rangle$	$\langle 0.86,0.23\rangle$	$\langle 0.60,0.13\rangle$	$\langle 0.46,0.56\rangle$	$\langle 0.64,0.33\rangle$
${a}_{2}$	$\langle 0.61,0.14\rangle$	$\langle 0.69,0.40\rangle$	$\langle 0.70,0.21\rangle$	$\langle 0.64,0.24\rangle$	$\langle 0.54,0.11\rangle$
${a}_{3}$	$\langle 0.82,0.13\rangle$	$\langle 0.70,0.12\rangle$	$\langle 0.70,0.35\rangle$	$\langle 0.73,0.20\rangle$	$\langle 0.89,0.25\rangle$
${a}_{4}$	$\langle 0.74,0.32\rangle$	$\langle 0.69,0.13\rangle$	$\langle 0.57,0.12\rangle$	$\langle 0.67,0.30\rangle$	$\langle 0.79,0.23\rangle$

**Table 8:** Weights of the criteria

	${c}_{1}$	${c}_{2}$	${c}_{3}$	${c}_{4}$	${c}_{5}$
${a}_{1}$	0.35	0.24	0.21	0.14	0.06
${a}_{2}$	0.38	0.25	0.17	0.12	0.08
${a}_{3}$	0.32	0.26	0.19	0.13	0.10
${a}_{4}$	0.35	0.26	0.19	0.12	0.08

Step 4: In the PF-RS approach, the aggregate values of alternatives can be calculated by Eq. (15). Then score values and rankings of alternatives are given in Table 9.

**Table 9:** Ranking by the PF-RS approach

	${y}_{i}$	${S}_{1}\left({y}_{i}\right)$	Ranking
${a}_{1}$	$\langle 0.7628,0.1815\rangle$	0.7744	2
${a}_{2}$	$\langle 0.6525,0.1565\rangle$	0.7006	4
${a}_{3}$	$\langle 0.7978,0.1416\rangle$	0.8082	1
${a}_{4}$	$\langle 0.7095,0.1521\rangle$	0.7401	3

Step 5: In the PF-RF approach, the reference points can be calculated based on Eq. (17), and the corresponding Pythagorean fuzzy sets are shown in Table 10. Distances from each alternative to all the coordinates of the reference point were calculated by Eq. (18), and the final ranking is presented in Table 11.

**Table 10:** Reference point

	${c}_{1}$	${c}_{2}$	${c}_{3}$	${c}_{4}$	${c}_{5}$
Reference point	$\langle 0.82,0.13\rangle$	$\langle 0.86,0.12\rangle$	$\langle 0.7,0.12\rangle$	$\langle 0.73,0.2\rangle$	$\langle 0.89,0.11\rangle$

**Table 11:** Ranking by the PF-RP approach

	${c}_{1}$	${c}_{2}$	${c}_{3}$	${c}_{4}$	${c}_{5}$	${S}_{2}\left({y}_{i}\right)$	Ranking
${a}_{1}$	0.2100	0.0385	0.1300	0.3213	0.3825	0.3825	3
${a}_{2}$	0.3003	0.2635	0.0297	0.1233	0.5005	0.5005	4
${a}_{3}$	0	0.2496	0.1081	0	0.0504	0.2496	1
${a}_{4}$	0.1248	0.2635	0.1651	0.084	0.1680	0.2635	2

Step 6: In the PF-FMF approach, the aggregate values of alternatives can be calculated by Eq. (20). Then score values and rankings of alternatives are given in Table 12.

**Table 12:** Ranking by the PF-FMF approach

	${y}_{i}$	${S}_{3}\left({y}_{i}\right)$	Ranking
${a}_{1}$	$\langle 0.6119,0.3253\rangle$	0.6343	4
${a}_{2}$	$\langle 0.6333,0.2631\rangle$	0.6660	3
${a}_{3}$	$\langle 0.7471,0.2167\rangle$	0.7556	1
${a}_{4}$	$\langle 0.6725,0.2472\rangle$	0.6956	2

Step 7: Using Eq. (22), the three MULTIMOORA subsystem ranking score values were normalized as follows:

$${S}_{\zeta }^{\mathrm{^{\prime}}}\left({y}_{i}\right)=\left[\begin{array}{ccc}0.5116& 0.5261& 0.4600\\ 0.4628& 0.6884& 0.4831\\ 0.5339& 0.3433& 0.5481\\ 0.4889& 0.3624& 0.5046\end{array}\right]$$

**Fig. 3:**
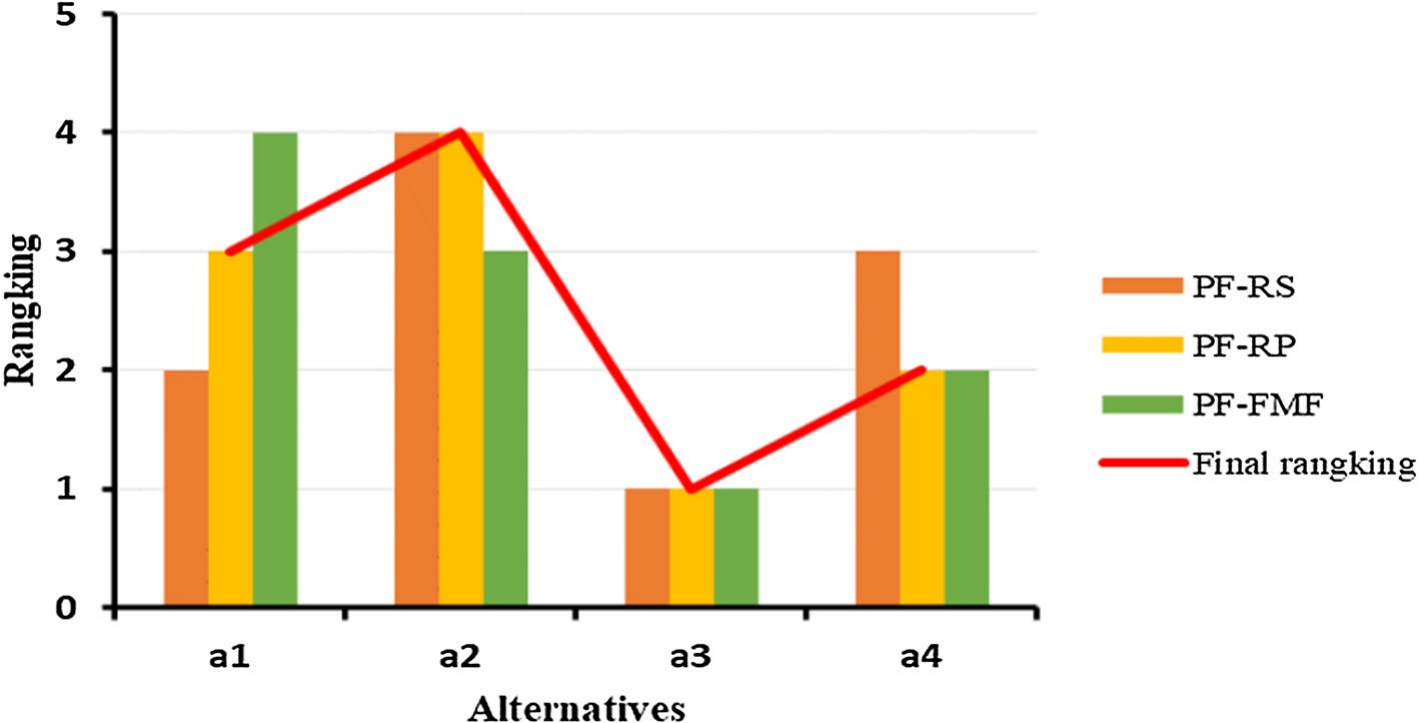
Final ranking of the alternatives based on the Borda scores

Then based on ${S}_{\zeta }^{\mathrm{^{\prime}}}\left({y}_{i}\right)$, the Borda scores ${\Psi }_{i}$ of alternatives were calculated by Eq. (23), the final ranking was calculated and is shown in Table 13, the visual ranking result is shown in Fig. 3.

**Table 13:** Final ranking of the alternatives based on the Borda scores

	${\rho }_{1}\left({y}_{i}\right)$	${\rho }_{2}\left({y}_{i}\right)$	${\rho }_{3}\left({y}_{i}\right)$	${\Psi }_{i}$	Ranking
${a}_{1}$	2	3	4	0.0416	3
${a}_{2}$	4	4	3	− 0.1325	4
${a}_{3}$	1	1	1	0.3985	1
${a}_{4}$	3	2	2	0.1767	2

In summary, the final ranking order of the alternatives is ${a}_{3}\succ {a}_{4}\succ {a}_{1}\succ {a}_{2}$. The proposed method determined “The Tarim Basin (${a}_{3}$)” as the best site for CO_2_ geological storage in China. Next, we mainly explore the impact of the parameter $\eta$ on the proposed model and compare our proposed model with the other Pythagorean fuzzy MCDM methods to highlight their effectiveness.

### Sensitivity Analysis

**Fig. 4:**
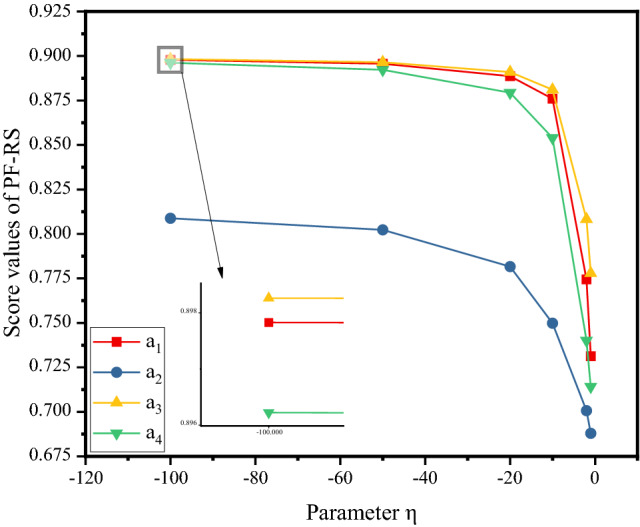
Score values of the alternative using PF-RS for different η

**Fig. 5:**
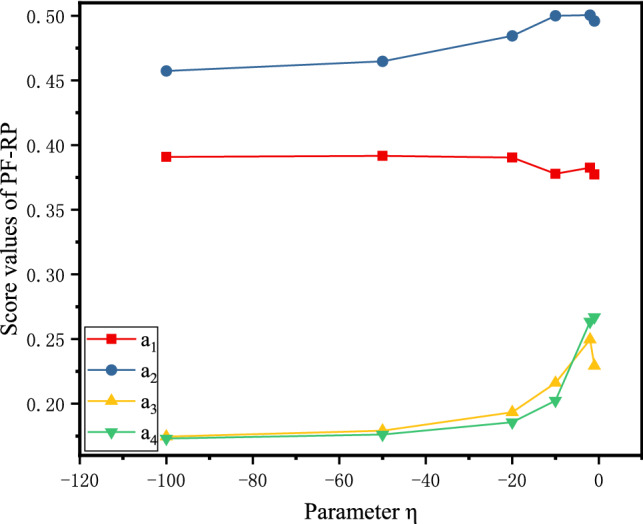
Score values of the alternative using PF-RP for different η

**Fig. 6:**
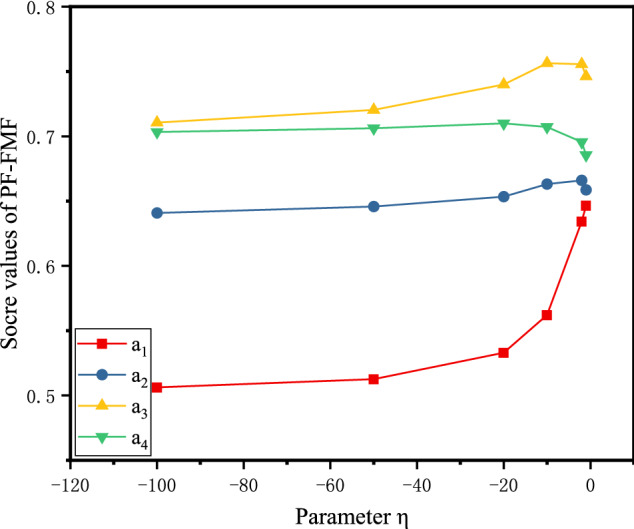
Score values of the alternative using PF-FMF for different η

**Fig. 7:**
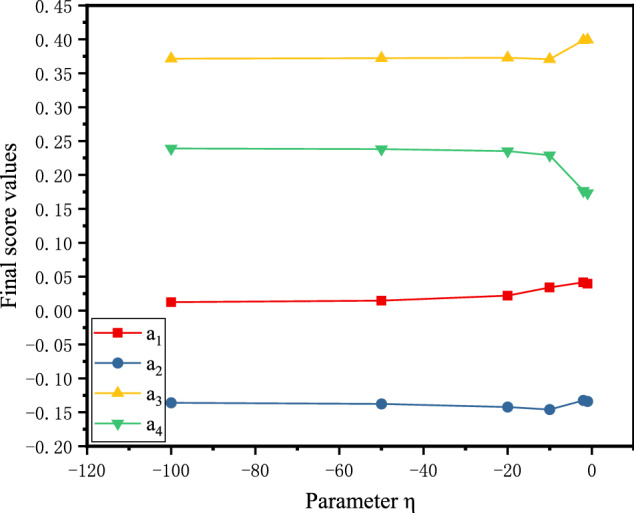
Score values of the alternative using the improved Borda rule for different η

From the definition of the PFSSPW operator, it can be seen that the decision-making process based on the PFSSPWA operator and PFSSPWG operator varies with the parameter $\eta$. To understand the performance of aggregation in depth, we adopt the parameter $\eta =-1,-10,-20,-50,-100$ for the numerical example below. When the parameters $\eta$ take different values, the score values can be obtained as shown in Figs. 4, 5, and 6 by PF-RS, PF-RP, and PF-FMF. Then the final score values are shown in Fig. 7.

In Fig. 4, for the PF-RS method, it is necessary to notice that the graphs corresponding to the score values of the alternatives are monotonically decreasing. This demonstrates that the higher value of the parameter $\eta$ must be assigned for making pessimistic decisions, whereas for making optimistic decisions, the decision maker selects a smaller value of the parameter $\eta$. In Fig. 5, for the PF-RP method, if $\eta =-1$, then ${a}_{3}\succ {a}_{4}$; if $\eta =-10$, then ${a}_{4}\succ {a}_{3}$, and the smaller the value of the parameter $\eta$ is, the smaller the difference between ${a}_{3}$ and ${a}_{4}$. In Fig. 6, for the PF-FMF method, the smaller the value of the parameter $\eta$ is, the smaller the score value of ${a}_{1}$. The score values of ${a}_{2},{a}_{3},{a}_{4}$ have minor fluctuations with the parameter $\eta$. In Fig. 7, it can be seen that the final ranking of alternatives is fixed no matter how the values of η are changed in the example, and the consistent ranking results demonstrate the robustness of the proposed approach.

### Comparison Analysis

**Fig. 8:**
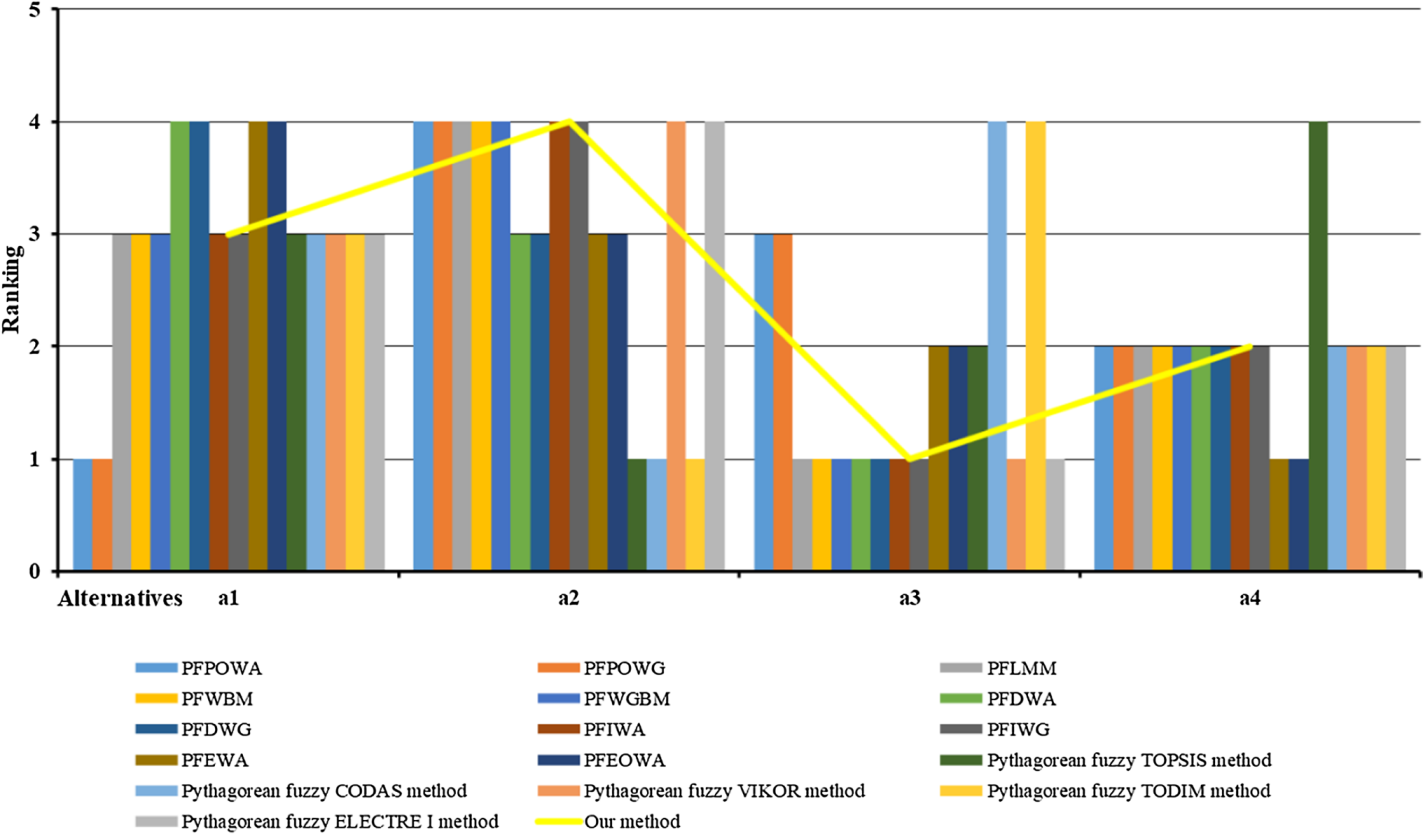
Comparison of alternative rankings with different Pythagorean fuzzy MCDM methods

In this section, the proposed method is compared with other Pythagorean fuzzy MCDM methods to further validate the flexibility and rationality. The ranking results of the selected methods are shown in Table 14. To provide a more visual comparison, the results of Table 14 are represented using a histogram, as shown in Fig. 8.

**Table 14:** Comparative analysis of the ranking results of different Pythagorean fuzzy MCDM methods

Methods	Ranking
PFPOWA operator [31]	${a}_{1}\succ {a}_{4}\succ {a}_{3}\succ {a}_{2}$
PFPOWG operator [31]	${a}_{1}\succ {a}_{4}\succ {a}_{3}\succ {a}_{2}$
PFLMM operator [32]	${a}_{3}\succ {a}_{4}\succ {a}_{1}\succ {a}_{2}$
PFWBM operator [33]	${a}_{3}\succ {a}_{4}\succ {a}_{1}\succ {a}_{2}$
PFWGBM operator [33]	${a}_{3}\succ {a}_{4}\succ {a}_{1}\succ {a}_{2}$
PFDWA operator [57]	${a}_{4}\succ {a}_{3}\succ {a}_{1}\succ {a}_{2}$
PFDWG operator [57]	${a}_{4}\succ {a}_{3}\succ {a}_{1}\succ {a}_{2}$
PFIWA operator [58]	${a}_{3}\succ {a}_{4}\succ {a}_{1}\succ {a}_{2}$
PFIWG operator [58]	${a}_{3}\succ {a}_{4}\succ {a}_{1}\succ {a}_{2}$
PFEWA operator [59]	${a}_{4}\succ {a}_{3}\succ {a}_{2}\succ {a}_{1}$
PFEOWA operator [59]	${a}_{4}\succ {a}_{3}\succ {a}_{2}\succ {a}_{1}$
Pythagorean fuzzy TOPSIS method [55]	${a}_{3}\succ {a}_{1}\succ {a}_{2}\succ {a}_{4}$
Pythagorean fuzzy CODAS method [60]	${a}_{3}\succ {a}_{1}\succ {a}_{4}\succ {a}_{2}$
Pythagorean fuzzy VIKOR method [61]	${a}_{3}\succ {a}_{4}\succ {a}_{1}\succ {a}_{2}$
Pythagorean fuzzy TODIM method [62]	${a}_{3}\succ {a}_{4}\succ {a}_{1}\succ {a}_{2}$
Pythagorean fuzzy ELECTRE I method [63]	${a}_{3}\succ {a}_{4}\succ {a}_{1}\succ {a}_{2}$
Our method	${a}_{3}\succ {a}_{4}\succ {a}_{1}\succ {a}_{2}$

It can be seen that the ranking results obtained using the PFLMM operator [32], the PFWBM operator [33], and the PFWGBM operator [33] are the same as those derived by our proposed method. This illustrates the feasibility and effectiveness of the proposed method. However, the results using the PFPOWA operator [31] and the PFPOWG operator [31] are completely different from our method. That is because our method is based on Schweizer-Sklar t-norm and t-conorm operations by adding an intrinsic parameter in both aggregation operators and may lead to reasonable conclusions.

An MCDM method based on the Pythagorean fuzzy Dombi weighted average (PFDWA) operator and Pythagorean fuzzy Dombi weighted geometric (PFDWG) operator [57], a better expression of application within the general parameter, was created by Jana. With the general parameter set to 1, the weights of the criteria are equal, and the ranking result is ${a}_{4}\succ {a}_{3}\succ {a}_{1}\succ {a}_{2}$. The best alternatives proposed by this method are different from those in our method, but the worst one is the same as ours.

Considering the interaction between the membership and non-membership functions in a Pythagorean fuzzy environment, Wei developed the Pythagorean fuzzy interaction weighted average (PFIWA) operator and the Pythagorean fuzzy interaction weighted geometric (PFIWG) operator [58] on the basis of traditional arithmetic and geometric operations. The ranking results obtained using this method are identical to those obtained using our method. So, the methods in this paper are effective and feasible. However, Wei’s method only considers the existence of interaction in PFNs, while our method considers the levels of priority in the criteria. Therefore, our method can more flexibly reflect the uncertainty in the decision-making process.

The Pythagorean fuzzy Einstein weighted average (PFEWA) operator and Pythagorean fuzzy Einstein ordered weighted average (PFEOWA) operator [59] were presented by Garg and extended the notion of aggregating the different PFNs using Einstein t-norm and t-conorm operations. The ranking results obtained using this method are different from those obtained using our method. In addition, the calculation process for this method is more complicated. Furthermore, Garg's methods do not take into account the interconnection of the input arguments, whereas the method proposed here can simulate the relationship between criteria; thus, our method is more realistic.

The Pythagorean fuzzy technique for order preference by similarity to an ideal solution (TOPSIS) method [55] compared to our method, the best alternative is always ${a}_{3}$, despite the different ranking of alternatives ${a}_{1}$, ${a}_{2}$ and ${a}_{4}$. The main idea of the Pythagorean fuzzy TOPSIS method is that the best alternative is the one that is closest to the positive ideal solution and farthest from the negative one. In addition, the Pythagorean fuzzy TOPSIS method uses the weighted average operator to aggregate the performances of alternatives, resulting in different ranking results.

An algorithm for solving the MCDM problem based on combinative distance-based assessment (CODAS) in a Pythagorean fuzzy environment was proposed by Peng [60]. The best alternative and the worst alternative proposed by this method are the same as those in our method, namely, ${a}_{3}$ is the best choice, and ${a}_{1}$ is the worst choice. However, the ranking results of ${a}_{1}$ and ${a}_{4}$ are different. The reason for this divergence is that Peng’s method used the Pythagorean fuzzy Euclidean distance measure and a novel score function for PFNs to judge the gap between each alternative and the negative ideal point. In our method, the rankings of the three subsystems (PF-RS, PF-RP, and PF-FMF) are combined to obtain the final ranking. Thus, our method is more representative.

The ordering position of the alternatives obtained using Gul’s method [61], based on the Pythagorean fuzzy VlseKriterijumska Optimizacija I Kompromisno Resenje (VIKOR) method, is identical to the proposed approach results. The essence of the VIKOR method is a compromise ideal: to find the relationship between the group utility values and the individual utility values. But due to the complex relationship between them, the ranks of each alternative are often difficult to determine, and there are certain drawbacks, making this method less robust.

The rank results derived by the proposed framework have some distinctions with those by the Pythagorean fuzzy TODIM method [62] and Pythagorean fuzzy ELECTRE I method [63]. The first reason is the different decision mechanisms. The Pythagorean fuzzy TODIM method and Pythagorean fuzzy ELECTRE I method ignore the priority of criteria; they give inaccurate results in complex situations. The second reason is the diverse aggregation mechanisms. This paper uses the PFSSPA operator to aggregate the group evaluation information, and considers the interactions among risk preferences.

Moreover, to verify the effectiveness of the final results, Spearman’s rank correlation test is conducted to analyze the relationship between the rankings of the 16 compared methods. The results show that there are eight methods in which Spearman’s rank correlation is 1, and the average value is 0.65. These verify the strong reliability between the existing MADM method and the proposed model.

From the above discussion, compared with the existing MCDM method in the Pythagorean fuzzy environment, the proposed method has the following advantages:

1. The model uses the membership and non-membership of the PFSs to express the fuzzy information of the experts. The vagueness of the original information will be maintained, which avoids information loss and distortion in the decision-making analysis process. As a result, the model can express and infer more about uncertain information.
2. The PFSSPWA and PFSSPWG operators involving variable parameters are more flexible than other existing operators. Decision makers can choose appropriate parameters to reflect their risk preferences, and the ranking results have reliability.
3. The proposed method in this article considers the relevance of the priorities between the criteria and avoids the influence of unknown weights on the decision-making results. Therefore, the concept of priority has an important role in our study.
4. Our developed PFSSPA-MULTIMOORA incorporated the advantages of three subsystems, and the final results gathered using the improved Borda rule made the ranking results more in line with the actual situation.

## Conclusion

This paper presents an extended fuzzy MULTIMOORA model based on the Pythagorean fuzzy sets and the Schweizer–Sklar t-norm and t-conorm for solving the site selection problem of CO_2_ geological storage. The proposed Pythagorean fuzzy MCDM model is composed of three main phases. First, the description and establishment of the problem involve the identification of criteria, alternatives, and experts. Second, determination of weights using decision matrix information and expert information aggregation using the proposed operator. Third, the PFSSPA-MULTIMOORA method is applied to rank the alternatives and select the optimal one. The main findings and achievements of this paper include the following five aspects: (1) The PFSs are used to express the complicated and uncertain evaluation data. The larger membership space of the PFS allows the proposed model to more effectively represent the decision information in the MCDM process. (2) Based on the Schweizer–Sklar t-norm and t-conorm, the PFSSPWA and PFSSPWG proposed in this paper contain adjustable parameters, and decision makers can choose appropriate parameters according to their own risk preferences. (3) Experts determine the different priority orders among criteria based on their own expertise and domain experience and use the PFSSPWA and PFSSPWG operators to aggravate the evaluation information among experts, which solves the MCDM problem of completely unknown criterion weights. (4) Three aggregation models (PF-RS, PF-RP, and PF-FMF) with different functions are utilized to handle the decision matrix and make full use of both aggregation operators and distance measurement. As a result, the proposed method can provide advanced decision support for researchers and practitioners. (5) The proposed method can be a useful tool for nations and regions choosing CO_2_ geological storage sites and dealing with other low-carbon technological issues. CO_2_ geological storage is the core component of CCUS technology, and the establishment of a decision model applicable to CO_2_ storage location selection determines the development potential and direction of CCUS technology.

Although the proposed model has the above-mentioned advantages, there are some limitations that could be discussed, the main focus is on the size of the criteria system, computational complexity, expert consensus, etc. First, considering the limitation of space and the complexity of the calculation, only five representative criteria are incorporated in this study. Second, the proposed method's complex calculations limit its use in some practical problems. Third, the proposed model does not take into consideration the achievement of expert consensus.

To address the above research limitations, the following areas of study might be explored in future work. (1) Future research should take into account more criteria to improve the decision-making technologies. (2) Designing a software tool to reduce the computational burden is a desirable route for future studies. (3) In real life, decision makers may offer varying judgments and evaluation information due to their varied backgrounds in education and experience. Therefore, the adjustment of expert opinions and the reach of expert consensus will be discussed in the further study.

## Data Availability

The manuscript contains all of the datasets that were used or analyzed for the current study.

## Abbreviations

CCUS: Carbon capture, utilization, and storage
MCDM: Multi-criteria decision-making
MULTIMOORA: Multi-objective optimization by ratio analysis plus the full multiplicative form
PFSSPA: Pythagorean fuzzy Schweizer–Sklar prioritized aggregation
PFSSPA-MULTIMOORA: Multi-objective optimization by ratio analysis plus the full multiplicative form method based on Pythagorean fuzzy Schweizer–Sklar prioritized aggregation operators
CO_2_: Carbon dioxide
PFSs: Pythagorean fuzzy sets
PA: Prioritized average
PFPOWA: Pythagorean fuzzy power ordered weighted average
PFPOWG: Pythagorean fuzzy power ordered weighted geometric
PFLMM: Pythagorean fuzzy linguistic Muirhead mean
PFWBM: Pythagorean fuzzy weighted Bonferroni mean
PFWGBM: Pythagorean fuzzy weighted geometric Bonferroni mean
PFSSPWA: Pythagorean fuzzy Schweizer–Sklar prioritized weighted average
PFSSPWG: Pythagorean fuzzy Schweizer–Sklar prioritized weighted geometric
PFNs: Pythagorean fuzzy number
PF-RS: Pythagorean fuzzy ratio system
PF-RP: Pythagorean fuzzy reference point
PF-FMF: Pythagorean fuzzy full multiplicative form
PFDWA: Pythagorean fuzzy Dombi weighted average
PFDWG: Pythagorean fuzzy Dombi weighted geometric
PFIWA: Pythagorean fuzzy interaction weighted average
PFIWG: Pythagorean fuzzy interaction weighted geometric
PFEWA: Pythagorean fuzzy Einstein weighted average
PFEOWA: Pythagorean fuzzy Einstein ordered weighted average
TOPSIS: Technique for order preference by similarity to an ideal solution
CODAS: Combinative distance-based assessment
VIKOR: VlseKriterijumska Optimizacija I Kompromisno Resenje
